# Analysis of lipid uptake, storage, and fatty acid oxidation by group 2 innate lymphoid cells

**DOI:** 10.3389/fimmu.2024.1493848

**Published:** 2024-10-21

**Authors:** Audrey Roy-Dorval, Rebecca C. Deagle, Frederik Roth, Mathilde Raybaud, Nailya Ismailova, Sai Sakktee Krisna, Damon G. K. Aboud, Camille Stegen, Julien Leconte, Gabriel Berberi, Ademola Esomojumi, Jörg H. Fritz

**Affiliations:** ^1^ Department of Microbiology and Immunology, McGill University, Montréal, QC, Canada; ^2^ McGill University Research Center on Complex Traits (MRCCT), McGill University, Montréal, QC, Canada; ^3^ Dahdaleh Institute of Genomic Medicine (DIgM), McGill University, Montréal, QC, Canada; ^4^ Department of Physiology, McGill University, Montréal, QC, Canada; ^5^ Department of Chemical Engineering, McGill University, Montréal, QC, Canada

**Keywords:** group 2 innate lymphoid cells (ILC2), type 2 immunity, immunometabolism, fatty acid uptake, lipid droplets, fatty acid oxidation (FAO), microscopy, flow cytometry

## Abstract

Group 2 Innate Lymphoid Cells (ILC2) are critical drivers of both innate and adaptive type 2 immune responses, known to orchestrate processes involved in tissue restoration and wound healing. In addition, ILC2 have been implicated in chronic inflammatory barrier disorders in type 2 immunopathologies such as allergic rhinitis and asthma. ILC2 in the context of allergen-driven airway inflammation have recently been shown to influence local and systemic metabolism, as well as being rich in lipid-storing organelles called lipid droplets. However, mechanisms of ILC2 lipid anabolism and catabolism remain largely unknown and the impact of these metabolic processes in regulating ILC2 phenotypes and effector functions has not been extensively characterized. ILC2 phenotypes and effector functions are shaped by their metabolic status, and determining the metabolic requirements of ILC2 is critical in understanding their role in type 2 immune responses and their associated pathophysiology. We detail here a novel experimental method of implementing flow cytometry for large scale analysis of fatty acid uptake, storage of neutral lipids, and fatty acid oxidation in primary murine ILC2 with complementary morphological analysis of lipid storage using confocal microscopy. By combining flow cytometry and confocal microscopy, we can identify the metabolic lipid requirements for ILC2 functions as well as characterize the phenotype of lipid storage in ILC2. Linking lipid metabolism pathways to ILC2 phenotypes and effector functions is critical for the assessment of novel pharmaceutical strategies to regulate ILC2 functions in type 2 immunopathologies.

## 1 Introduction

Group 2 innate lymphoid cells (ILC2) instruct innate type 2 immune responses, exerting critical roles in the initiation and orchestration of anti-helminth immunity as well as allergic inflammation (1–3). ILC2 share functional overlap with antigen-specific CD4^+^ type 2 T helper (Th2) cells, including the requirement of the transcriptional regulator GATA3 as well as the release of type 2 signature cytokines such as interleukin (IL)-4, IL-5 and IL-13, facilitating eosinophil recruitment, goblet cell hyperplasia and mucus production (4, 5). ILC2 are located at internal and external barrier surfaces including the lung (6). However, in contrast to Th2 cells, ILC2 lack the expression of specific antigen receptors and are primarily activated in an antigen-independent fashion in response to alarmins such as IL-33, IL-25 and/or thymic stromal lymphopoietin (TSLP) that are released upon tissue perturbation or immune challenge (4, 5). IL-33 has been described as the most potent activator of lung ILC2 (7), signaling through the heterodimeric IL-33 receptor (IL-33R) composed of the ligand-binding chain ST2 and IL-1 receptor accessory protein (IL-1RacP) (8, 9). Furthermore, it was recently demonstrated that ILC2 require cell-intrinsic ST2 signals to promote type 2 responses, including allergic airway inflammation (10).

Due to the potency to rapidly release type 2 signature cytokines such as IL-4, IL-5 and IL-13 upon activation, ILC2 have been suggested to constitute a critical therapeutic target to treat human type 2 immunopathologies (11). Indeed, elevated frequencies and numbers of ILC2 have been found in patients with asthma, allergic rhinitis and chronic rhinosinusitis, correlating with disease severity and resistance to corticosteroid therapy (12, 13). Biologics, including mepolizumab, benralizumab, and dupilumab, targeting cytokines IL-5, and IL-4/IL-13, respectively, have shown promising effects, leading to the reduction in annualized asthma exacerbation rates (AER), oral corticosteroid-sparing effects and improved Asthma Control Questionnaire scores (14, 15). However, despite these clinical advances, approximately 30% of patients with severe asthma receiving biologics do not experience meaningful improvements in their AER (14, 16). Hence, instead of blocking downstream type 2 signature cytokines, the targeting of upstream alarmins, including IL-33 and TSLP has been proposed as an alternative treatment strategy for asthma and type 2 immunopathologies (14, 15). Indeed, initial clinical trials targeting IL-33 using Itepekimab (17, 18) or TSLP through the application of Tezepelumab (19) show promising clinical results by reducing asthma symptoms. However, the cost of biologics is high and can place financial pressures on patients and health care systems, estimating the annual net price for each of the drugs at approximately $30,000 annually (20). In addition, asthma phenotypes are multi-layered and a better understanding of their diversity is needed to allow individualized treatment strategies for patients (14, 21).

Fueled by a wealth of mechanistic insights from studies in oncology and on metabolic disorders it has become increasingly clear that targeting the metabolism of immune responses can improve clinical treatment outcomes (22, 23). As such, a better understanding of the metabolic wiring of the diseased tissue and its cellular components is critical to understand strengths and limitations of current treatment strategies of type 2 immunpathologies. Indeed, metabolomic studies have revealed associations between altered lipid metabolism, amino acid metabolism and disease pathogenesis in asthmatic patients (24–26). Moreover, obesity was shown to be the most common comorbidity of asthma (27), revealing that energy metabolism is altered in obese asthmatics (28). As ILC2 have been shown to exert critical roles in the regulation of tissue as well as systemic metabolism (29) they constitute a critical target for metabolic intervention to treat type 2 immunopathologies.

While glucose and amino acid metabolism were shown to be important to sustain ILC2 proliferation and effector functions (29–31) it has recently become increasingly clear that uptake and metabolism of lipids also exerts critical metabolic functions in ILC2. During helminth infection and allergen-induced airway inflammation intestinal as well as lung ILC2 were found to increase their exogenous fatty acid (FA) intake, respectively (32–35). Moreover, it was observed that in addition to increasing exogenous lipid acquisition, lung ILC2 store more lipids in lipid droplets, facilitating ILC2 functions during allergen-induced airway inflammation (33, 35). In correlation with increased lipid droplets in ILC2, *in vivo* treatment with papain or with IL-33 increased DGAT1 gene expression (33), an enzyme involved in triacylglyceride (TAG) formation and LD biogenesis (36, 37). ILC2-specific deletion was found to reduce ILC2 effector function and allergic airway inflammation (33), demonstrating a critical role for LD biogenesis and DGAT1 in the regulation of ILC2 effector functions. FAs can be catabolized in mitochondria by fatty acid β-oxidation (FAO) (38). As a result, acetyl-CoA is produced and can help fuel numerous metabolic pathways including the tricarboxylic acid (TCA) cycle (38). In addition, FAO results in the reduction of flavin adenine dinucleotide (FAD) to FADH2 and nicotinamide adenine dinucleotide (NAD) to NADH, important electron donors to the respiratory chain (38). It has previously been reported that IL-33-stimulated ILC2 employ oxidative phosphorylation to a greater extent than steady state ILC2 (31). In fact, during helminth infection, it has been reported that ILC2 metabolize exogenous FA via FAO to fuel oxidative phosphorylation, which was necessary for ILC2 proliferation and cytokine production (32, 33). IL-33 treated lung-derived ILC2 also required FAO to produce cytokines and proliferate (39). Therefore, lipid uptake and lipid metabolism play an important role in ILC2 metabolism for proliferation and the regulation of effector functions. We thus aimed to develop a methodological workflow to enable rapid analysis of lipid uptake, lipid storage and fatty acid oxidation by primary ILC2.

## 2 Materials and methods

### 2.1 Mice

C57BL/6J wildtype (WT) mice were originally purchased from the Jackson Laboratory (Bar Harbor, ME) and bred in house at McGill University. All animals were maintained on a C57BL/6J background, bred, and housed under specific pathogen-free conditions with ad libitum access to food and water. All experiments were performed on female mice (aged 6-12 weeks) in accordance with the guidelines and policies of the Canadian Council on Animal Care and those of McGill University.

### 2.2 *In vivo* intranasal treatment for *ex vivo* experiments

**Figure 1:**
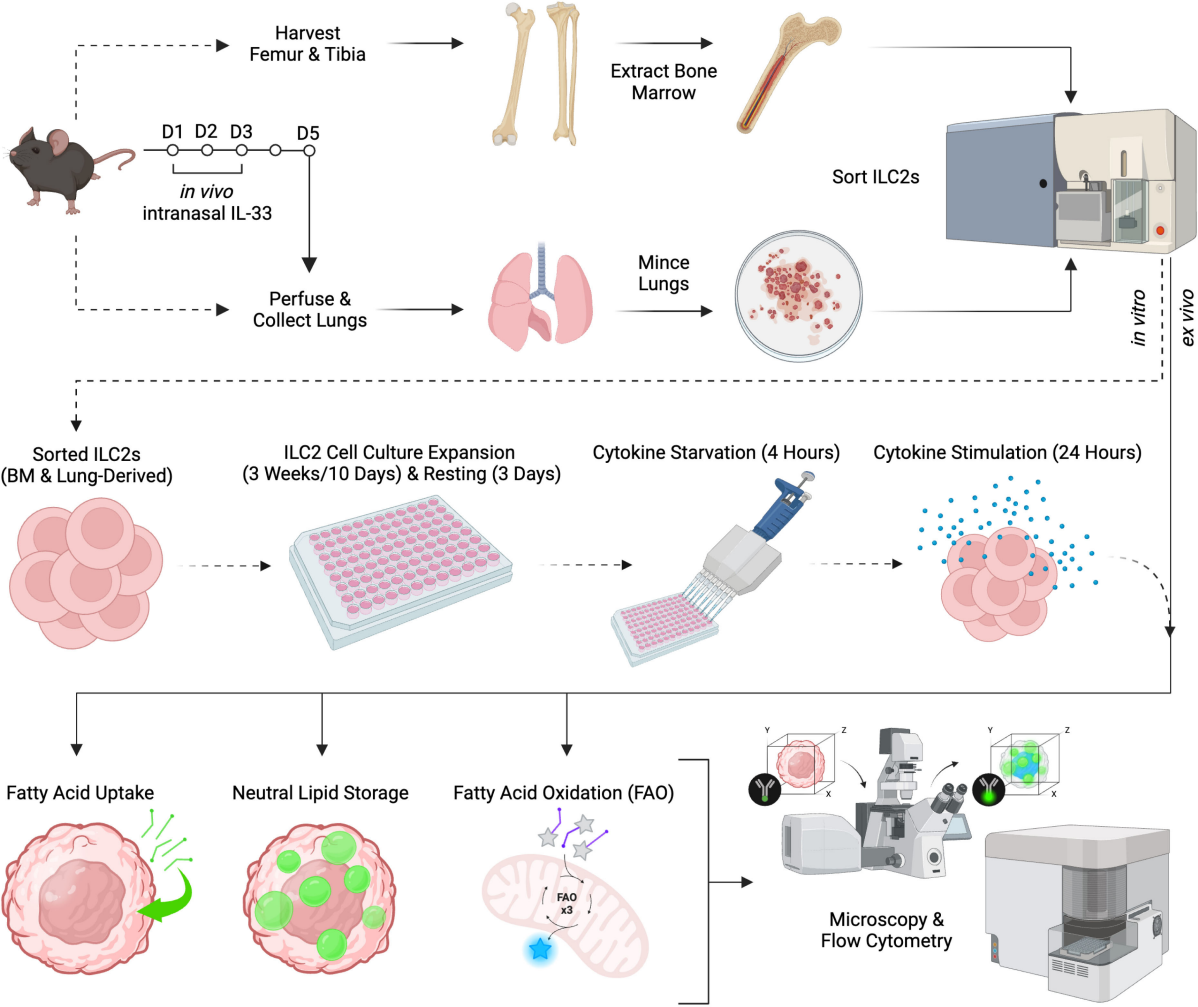
Schematic of experimental workflow. Female C57BL/6J wild-type mice aged 6-12 weeks were selected for either bone marrow or lung group 2 innate lymphoid cell (ILC2) isolation. Femurs and tibias were harvested to obtain bone marrow-derived ILC2 by flow cytometric cell sorting and were then cultured *in vitro* (dashed line) for expansion, followed by cytokine starvation and subsequent cytokine re-stimulation. Lung-derived ILC2 were obtained by flow cytometric cell sorting from naïve mice followed by *in vitro* cell culture expansion (dashed line). Alternatively, lung-derived ILC2 were obtained by flow cytometric cell sorting from animals that were challenged intranasally with PBS as control or IL-33 for three consecutive days (solid line). Bone marrow- and lung-derived ILC2 were used for the quantification of fatty acid uptake, neutral lipid storage, and fatty acid oxidation (FAO) by flow cytometry or microscopy. Created in BioRender.com. Deagle, R. (2024) BioRender.com/v17y111.

As previously reported (40), mice were anaesthetized with 5% Isoflurane USP (Fresenius Kabi, Catalog No. CP0406V2) and intranasally challenged with 250 ng of carrier-free recombinant murine IL-33 (R&D Systems, Catalog No. 3626-ML-010/CF) in 40 μL of Dulbecco’s Phosphate Buffered Saline (DPBS; Fisher Scientific, Catalog No. SH30028.02) or DPBS alone for three consecutive days. The 40 µL volume was divided, whereby 20 µL was administrated per nostril. On the fifth day, lungs were collected and isolated as outlined in Section 2.4 (**Figure 1**).

### 2.3 Isolation of bone marrow-derived ILC2

Mice were anaesthetized with 5% isoflurane and euthanized via CO_2_ asphyxiation, followed by cervical dislocation as confirmation of death. The femurs and tibias were collected, cleaned with sterilized gauze (Fisher Scientific, Catalog No. 22-037-907), and washed in 75% ethanol prior to bone marrow (BM) extraction. Sterile BM extraction tubes were prepared by using an 18-gauge needle (Becton Dickinson, Catalog No. 305196) to puncture a single hole in the bottom of a 0.5 mL tube (Sarstedt, Catalog No. 72.737.002). These tubes were then autoclaved and set inside a sterile 1.5 mL collection tube (Progene, Catalog No. 87-B150-C) containing 100 µL of DPBS. Both ends of the cleaned bones were cut at the epiphysis to expose the BM and a single bone was placed vertically inside each 0.5 mL tube. The 1.5 mL tubes were then sealed and centrifuged (1900*g, 5 min, 4°C), which allowed the BM to be extracted from the bone, through the punctured hole, and into the 1.5 mL tube containing DPBS. The 0.5 mL tubes containing the empty bones were discarded, and the DPBS was resuspended with the pellet of BM and then consolidated into a 50 mL tube (Fisher Scientific, Catalog No. 14-432-22) with a maximum of 20 bones (5 mice) per tube. Each tube was filled to 50 mL with DPBS, centrifuged (450*g, 5 min, 4°C), and the supernatant was discarded. The cell pellet was resuspended in 1.5 mL of Ammonium-Chloride-Potassium (ACK) lysing buffer (Thermo Scientific, Catalog No. A1049201) for approximately 20-30 seconds to lyse red blood cells within the sample. This reaction was neutralized immediately by filling the tube with fluorescence-activated cell sorting (FACS) buffer to 50 mL followed by centrifugation (450*g, 5 min, 4°C) and a second wash with FACS buffer (450*g, 5 min, 4°C). FACS buffer was made in-house using DPBS and 2% fetal bovine serum (FBS; Wisent Bioproducts, Catalog No. 080150). To prevent non-specific binding of antibodies to the fragment crystallization receptors (FcRs), the cells were blocked for 15 minutes on ice using a 1:10 dilution of in-house formulated “Fc-block” (supernatant of the 2.4G2 hybridoma producing the purified anti-mouse CD16/CD32 monoclonal antibody (mAb)) in FACS buffer. The BM cells were then stained for 30 minutes on ice in the dark with an antibody cocktail (**Table 1**). The BM cells were then washed twice in FACS buffer as described above and filtered through a 70 µm cell strainer (Fisher Scientific, Catalog No. 22-363-548) prior to cell sorting. ILC2 were sorted using a FACS Aria III and FACSAria Fusion (BD Biosciences) equipped with 405 nm, 488 nm, 561 nm (Fusion only) and 640 nm lasers using FACSDiva version 6.0 (Aria III) or version 8.0 (Fusion) based on the absence of lineage markers and the expression of ILC2 specific markers (**Table 1**; **Supplementary Figure 1** reference to isolation of BM and lung ILC2).

**Table 1:** Antibody staining panels for bone marrow- and lung-derived ILC2.

Target	Vendor	Catalog Number	Concentration (µg/mL)	RRID
*Lineage Cocktail (LC)*				
TCRβ	ThermoFisher Scientific	12-5961-83	0.50	AB_466067
TCRγδ	ThermoFisher Scientific	12-5711-82	0.25	AB_465934
CD3ϵ	ThermoFisher Scientific	12-0031-83	0.25	AB_465497
Gr-1	ThermoFisher Scientific	12-5931-83	0.20	AB_466046
CD11b	ThermoFisher Scientific	12-0112-83	0.20	AB_2734870
Ter-119	ThermoFisher Scientific	12-5921-83	0.25	AB_466043
B220	ThermoFisher Scientific	12-0452-83	0.50	AB_465672
CD19	ThermoFisher Scientific	12-0193-83	0.50	AB_657660
NK1.1	ThermoFisher Scientific	12-5941-83	2.00	AB_466051
CD5	ThermoFisher Scientific	12-0051-83	0.50	AB_465524
CD11c	ThermoFisher Scientific	12-0114-83	1.00	AB_465553
FcϵR1α	ThermoFisher Scientific	12-5898-83	1.00	AB_466029
*Bone marrow-derived ILC2*				
LC				
Sca-1	BioLegend	122516	1.25	AB_756201
CD25	ThermoFisher Scientific	48-0251-82	2.00	AB_10671550
c-kit	ThermoFisher Scientific	17-1171-83	4.00	AB_469431
*BODIPY staining of lung-derived ILC2*				
LC				
CD25	ThermoFisher Scientific	48-0251-82	1.00	AB_10671550
ST2	ThermoFisher Scientific	47-9335-82	2.00	AB_2848379
CD90.2	BioLegend	140319	0.17	AB_2561395
CD45	BioLegend	103149	0.50	AB_2564590
7-AAD (viability dye)	ThermoFisher Scientific	00-6993-50	0.84	NA
*FAO-Blue staining of lung-derived ILC2*				
LC				
CD25	ThermoFisher Scientific	53-0251-82	2.50	AB_763472
ST2	ThermoFisher Scientific	47-9335-82	2.00	AB_2848379
CD90.2	BD Biosciences	567736	0.33	NA
CD45 (*ex vivo*)	BioLegend	103114	0.50	NA
CD45 (*in vitro*)	BioLegend	103149	0.50	NA
7-AAD (viability dye)	ThermoFisher Scientific	00-6993-50	0.84	NA

### 2.4 Isolation of lung-derived ILC2

Mice were anaesthetized with 5% isoflurane and euthanized via CO_2_ asphyxiation, followed by cervical dislocation as confirmation of death. The mice were then bisected at the sternum to facilitate the puncturing of the thoracic diaphragm and removal of the ribcage to expose the lungs. A 23-gauge needle (Becton Dickinson, Catalog No. 305145) attached to a 10 mL syringe (Becton Dickinson, Catalog No. 302995) filled with DPBS was used to perfuse the lungs via the heart by piercing the apex of the left ventricle. After perfusion, the thymus and heart were discarded, and the lungs were removed as a whole unit to be kept in cell culture media (**Table 2**) on ice until all the mice were processed. The lungs were cleaned using sterile gauze, and each lobe was separated from its connective tissue before being placed into a dry, sterile 6-well plate (VWR, Catalog No. 62406-161) with a maximum of 5 lungs per well. The lungs were mechanically minced to a fine paste using scissors, and 5 mL of cell culture media (**Table 2**) containing DNase (Roche, Catalog No. 10104159001) and Liberase (Milipore Sigma, Catalog No. 5401127001) with a final concentration of 100 µg/mL and 200 µg/mL, respectively, were added to each well for enzymatic digestion. The lung tissues were incubated with the enzymes at 37°C in 5% CO_2_ for a total of 30 minutes, stirring the plate at 10 minute intervals. To further homogenize the tissue, an 18-gauge needle attached to a 5 mL syringe (Becton Dickinson, Catalog No. 309646) was used to aspirate and dispense the tissue suspension a maximum of 3 times to prevent excessive shear-stress. The tissue suspension was then transferred to a 70 µm cell-strainer set inside a 50 mL tube for cell collection. The plunger of the 5 mL syringe was removed and the blunt, rubber end was used to mechanically dissolve the remaining pieces of lung tissue in the cell-strainer. The blunt end of the plunger and the 6-well plate were washed frequently with DPBS and added to the cell-strainer to maximize the cell yield. The 50 mL tubes were filled with DPBS to 50 mL and centrifuged (450*g, 5 min, 4°C) followed by a second wash of DPBS (450*g, 5 min, 4°C). The procedure for lysing red blood cells, blocking, antibody staining, and filtration followed the exact same guidelines as the BM-derived ILC2 isolation outlined in Section 2.3. Lung-derived ILC2 were then sorted purified using a BD FACS Aria III and BD FACSAria Fusion based on the absence of lineage markers and the expression of lung ILC2 specific markers (**Table 1**; **Supplementary Figure 1** for isolation of BM and lung ILC2).

**Table 2:** Complete RPMI media recipes for bone marrow- and lung-derived ILC2.

Component	Complete Cell Culture Media	FAO-Blue Staining Solution
RPMI 1640 Media without L-Glutamine	45 mL *With Phenol Red: Cytiva*, *Catalog No. SH30096.02;* *Phenol Red-Free: Cytiva*, *Catalog No. SH30605.01*	5 mL *(Phenol Red-Free)*
Fetal Bovine Serum (FBS), Heat inactivated *Wisent Bioproducts*, *Catalog No. 080150*	5 mL (10%)	100 µL (2%)
L-Glutamine (200mM) *Cytiva, Catalog. No. SH30034.01*	0.5 mL (2000 µM)	50 µL (2000 µM)
Penicillin/Streptomycin (10,000 U/mL/10,000 ug/mL) *Cytiva, Catalog No. SV30010*	0.5 mL (100 U/mL/100 µg/mL)	0 µL
Gentamycin (10 mg/mL) *Sigma Aldrich*, *Catalog No. G1272-100ML*	120 µL (24 µg/mL)	0 µL
β-mercaptoethanol (55mM) *Gibco*, *Catalog No.* 21-985-023	50 µL (55 µM) *Omitted for resting and experiments*	0 µL

### 2.5 *In vitro* expansion and resting of bone marrow and lung-derived ILC2

Sorted ILC2 were cultured in 96-well round-bottom plates (VWR, Catalog No. CA62406-121) at 37°C in 5% CO_2_ with a seeding density of 2.5x10^4^ cells per well in 200 µL of complete ILC2 cell culture media (**Table 2**) (40, 41). For BM-derived ILC2, 50 ng/mL of IL-2 (R&D Systems, Catalog No. 402-ML-100/CF), IL-7 (R&D Systems, Catalog No. 407-ML-200/CF), and IL-33 (R&D Systems, Catalog No. 3626-ML-010/CF) as well as 20 ng/mL of TSLP (R&D Systems, Catalog No. 555-TS-010/CF) were added to the cell culture media to promote the survival and expansion of the ILC2 *in vitro* cell culture. For lung-derived ILC2, 50 ng/mL each of IL-2 and IL-7 were added to the complete cell culture media for *in vitro* expansion. Phenol-red free complete media (**Table 2**) was specifically used to culture cells for fatty acid oxidation (FAO) experiments (Section 2.11) to avoid auto-fluorescent interference. Every 2 days the cell culture was split; whereby each well of ILC2 were resuspended and 100µL of cell suspension was transferred to an empty well and 100 µL of complete media with expansion cytokines were added to all wells containing ILC2 for a total volume of 200 µL. BM-derived ILC2 expanded *in vitro* for up to 3 weeks whereas lung-derived ILC2 were expanded *in vitro* for up to 10 days. BM- and lung-derived ILC2 intended for *ex vivo* experiments were used immediately after the sorting process and did not undergo *in vitro* expansion. After expansion was complete, the ILC2 were pooled, the expansion media was removed, and the cells were washed in RPMI 1640 (450*g, 5 min, 4°C) to remove any trace of expansion cytokines. The ILC2 were then re-seeded at 2.5x10^4^ cells per well for 3 days at 37°C in 5% CO_2_ in complete media containing 10 ng/mL of IL-2 and IL-7 to “rest” the cells. This period of reduced activity, or rest, was necessary to bring the ILC2 down to a homeostatic baseline level of activity (proliferation & cytokine production) after the demands of expansion.

### 2.6 ILC2 seeding and stimulation for flow cytometric assays and microscopy

After 3 days of resting, *in vitro* ILC2 were pooled together in a 50 mL tube and washed twice with RPMI 1640 media (450*g, 5 min, 4°C) to remove any trace of resting cytokines. *In vitro* ILC2 were resuspended in complete cell culture media (**Table 2**), counted, and the cell concentration was adjusted to 500,000 cells/mL. For flow cytometric assays, 100 µl of *in vitro* ILC2 (5x10^4^ cells) were seeded per well in a sterile 96-well round-bottom culture plate, whereas for microscopy, 1 mL of *in vitro* ILC2 (5x10^5^ cells) were seeded per well in a sterile 6-well plate. Seeded *in vitro* ILC2 incubated for 4 hours at 37°C in 5% CO_2_ to cytokine starve the cells, bringing the cells to a quiescent state prior to cytokine stimulation. The cytokine stimulations used for all *in vitro* experiments were the following: IL-7, IL-2, IL-33, IL-7+IL-33, and IL-2+IL-33. Each cytokine stimulation was made at a 2X concentration (20 ng/mL) in complete cell culture media and was added to the appropriate wells (100 μL for flow cytometry or 1 mL for microscopy) for a final 1X concentration (10 ng/mL). Cells were then incubated for 24 hours at 37°C in 5% CO_2_ prior to further analyses. *Ex vivo* ILC2 were seeded at the same density as described above after being retrieved from the cell sorter but did not undergo cytokine starvation or stimulation prior to further analyses.

### 2.7 Enzyme-linked immunosorbent assay

Production of IL-5 was quantified from supernatant of cultured murine bone marrow- and lung-derived ILC2 using the IL-5 mouse DuoSet ELISA kits (R&D Systems, Catalog No. DY405) according to the manufacturer’s instructions. Absorbance at 450 nm was measured using an Enspire™ 2300 Multilabel Reader (PerkinElmer).

### 2.8 Proliferation assay

Bone marrow ILC2 were stained with CellTrace Yellow Cell Proliferation Kit (Invitrogen, Catalog No. C34573) according to manufacturer’s instructions. Cells were plated at 20,000 cells/well with respective cytokines. After 3 days, ILC2 were stained with eFluor 780 Fixable Viability Dye (Invitrogen, Catalog No. 65-0865-18), fixed and permeabilized with FoxP3 Transcription Factor Staining Buffer Set (Invitrogen, Catalog No. 00-5523-00). Data were acquired using an Aurora Spectral Flow Cytometer (Cytek).

### 2.9 Flow cytometric staining of fatty acid uptake

The fatty acid (FA) uptake dye, BODIPY™ FL C16 (Invitrogen, Catalog No. D3821), was stored at -20°C at a stock concentration of 1 mM (1 mg stock powder in 2.11 mL DMSO) and diluted to an intermediate concentration of 5 µM. At the 23-hour mark of cytokine stimulation (*in vitro*) or after cell sorting (*ex vivo*), 50 μL of prepared 5 µM BODIPY™ FL C16 was added to each well to obtain a final working concentration of 1 μM and the cells were then incubated with the dye for 1 hour at 37°C in 5% CO_2_. After incubation, stimulated ILC2 were resuspended and transferred to a sterile 96-well conical-bottom culture plate (Sarstedt, Catalog No. 82.1583.001). The plate was then centrifuged (450*g, 5 min, 4 °C), the supernatant was removed, and cells were washed twice with 200 μL of FACS buffer. The supernatant was removed and the ILC2 were resuspended in 150 μL of FACS buffer, followed by an additional 5 μL of 7-AAD viability staining solution. All samples were immediately acquired by flow cytometry (Section 2.12).

### 2.10 Flow cytometric staining of neutral lipid content

The neutral lipid dye, BODIPY™ 493/503 (Invitrogen, Catalog No. D3922), was stored at -20°C at a stock concentration of 1.9 mM (10 mg stock powder in 1mL DMSO) and diluted to a working concentration of 2 µM in DPBS. After 24 hours of cytokine stimulation, ILC2 cultured *in vitro* were resuspended and transferred to a sterile 96-well conical-bottom culture plate. *Ex vivo* ILC2 were plated directly after sort purification into the sterile 96-well conical-bottom culture plate. The plate was then centrifuged (450*g, 5 min, 4 °C), the supernatant was removed, and the ILC2 were washed twice with 200 μL of cold DPBS (450*g, 5 min, 4 °C). After the supernatant was removed, the ILC2 were resuspended in 50 μL of prepared 2 μM BODIPY™ 493/503 and incubated for 20 minutes at 37°C in 5% CO_2_. After incubation, ILC2 were washed twice with 150 μL of FACS buffer (450*g, 5 min, 4 °C). The supernatant was removed and ILC2 were resuspended in 150 μL of FACS buffer in addition to 5 μL of 7-AAD viability staining solution. All samples were immediately acquired by flow cytometry (Section 2.12).

### 2.11 Flow cytometric staining of fatty acid oxidation

The fatty acid oxidation (FAO) detection dye, FAOBlue (DiagnoCine, Catalog No. FNK-FDV-0033) was stored at -20°C at a stock concentration of 1 mM (0.2 mg stock powder in 418.84 µL DMSO) and diluted to a working concentration of 20 µM in FAOBlue Staining Solution (**Table 2**). Throughout the staining procedure the FAOBlue dye as well as all the stained cells were kept on ice in the dark. After 24 hours of cytokine stimulation, ILC2 cultured *in vitro* were resuspended and transferred to a sterile 96-well conical-bottom culture plate. *Ex vivo* ILC2 were plated directly after sort purification into the sterile 96-well conical-bottom culture plate. The plate was centrifuged (450*g, 5 min, 4 °C), the supernatant was removed, and the ILC2 were washed twice with 200μL Phenol Red-Free RPMI 1640 (450*g, 5 min, 4 °C). The supernatant was removed and the cells were resuspended in 200 µL of the prepared 20 µM FAOBlue staining solution (**Table 2**). ILC2 were then incubated with the dye for 1 hour at 37°C in 5% CO_2_. The plate was centrifuged (450*g, 5 min, 4 °C), the supernatant was removed, and cells were washed twice with 200μL DPBS (450*g, 5min, 4°C). The supernatant was removed, and the cells were resuspended in 150 µL Phenol Red-Free RPMI 1640 and 5 µL of 7-AAD viability staining was added to each well immediately before acquiring data by flow cytometry (Section 2.12).

### 2.12 Flow cytometry analysis

**Figure 2:**
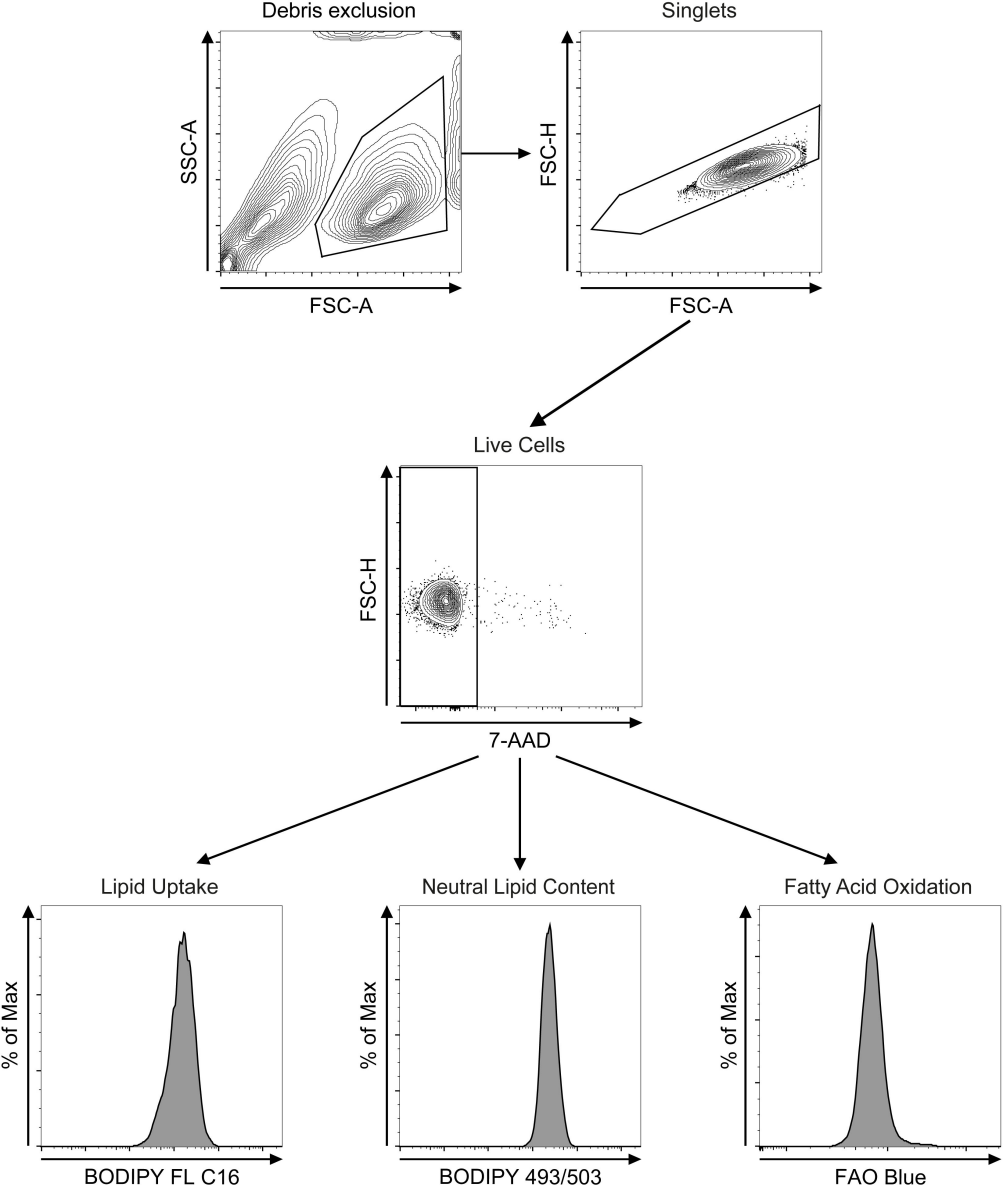
Flow cytometry gating strategies. After debris exclusion (FSC-A vs SSC-A) bone marrow- or lung-derived group 2 innate lymphoid cells (ILC2) were gated on singlets (FSC-A vs FSC-H) and on 7-AAD-negative live cells (7-AAD vs FSC-H). ILC2 were subsequently analyzed for lipid uptake (BODIPY FL C16), neutral lipid content (BODIPY 493/503) or fatty acid oxidation (FAO Blue).

Samples were acquired on a BD FACSCanto II (BD Biosciences) equipped with a 488 nm and a 633 nm laser and FACSDiva 8.0 (BODIPY) or on an Aurora Spectral Flow Cytometer (Cytek) (FAO Blue) equipped with 405 nm, 488 nm, 561 nm and 641 nm lasers and Spectroflo 2.0. Each sample was run until 20,000 events were recorded. All samples were gated on lymphocytes excluding debris, singlets, and dead ILC2 using 7-AAD viability staining following the manufacturer’s instructions. Then, FA uptake, neutral lipid content, or FAO were quantified by gating on BODIPY™ FL C16, BODIPY™ 493/503, and FAO Blue, respectively (**Figure 2**). Flow cytometry analysis was performed using FlowJo software (BD, Version 10.10.0). Geometric mean fluorescence intensity (MFI) of the signals was calculated, duplicates were averaged per stimulatory condition and presented in bar graphs with all flow cytometry data represented as mean ± standard deviation. Histograms and bar graphs were created using FlowJo and Prism softwares (Graphpad, Version 9) respectively. Statistical analysis was performed as ordinary one-way ANOVA and *post-hoc* Tukey’s multiple comparison tests to obtain statistical significance (P-values) between experimental conditions.

### 2.13 Staining of ILC2 for microscopy analyses

ILC2 were stained with the nuclear dye Hoechst 33342 (ThermoFisher Scientific, Catalog No. 62249), and with BODIPY™ 493/503 to visualize neutral lipid storage. Prior to staining, an intermediate concentration of Hoechst 33342 (stored at 4°C, stock concentration of 20 mM) was made by diluting 81.3 µl in 1 mL of DPBS for a concentration of 1 mg/mL. After 24 hours of cytokine stimulation (*in vitro*) or after cell sorting (*ex vivo*), 1 mL of cell culture media from each well was transferred to a 15 mL tube (Fisher Scientific, Catalog No. 14-959-49B). This media was used to make a 2X concentrated staining solution of Hoechst 33342 (3.25 µM) and BODIPY™ 493/503 (4 µM). The staining solution was thoroughly mixed and then added to each well, whereby the solution was pipetted slowly against the edge of the well to minimize cellular disturbance. The plate was gently tipped to ensure a homogenous distribution of the dye for a final working concentration of 1.6 µM of nuclear dye and 2 µM of neutral lipid dye. The cells were then incubated for 30 minutes at 37°C in 5% CO_2_.

After incubation, *in vitro* BM-derived ILC2 were resuspended and transferred to a 15 mL tube and centrifuged (450*g, 5min, 4°C). The supernatant was removed, and the cell pellet was resuspended in 1 mL of Hank’s Balanced Salt Solution (HBSS; ThermoFisher Scientific, Catalog No. 14175095) then transferred to a 1.5 mL tube and washed a second time with HBSS (450*g, 5 min, 4 °C). The supernatant was removed, and the cell pellet was resuspended in 500 µL of 4% formaldehyde fixative solution (Sigma-Aldrich, Catalog No. 252549) and incubated at room temperature for 10 minutes. After incubation, 1 mL of DPBS was added to each tube and centrifuged (450*g, 5 min, 4 °C) followed by a wash of DPBS with the same settings. After the supernatant was removed, the cell pellet was resuspended in 20 µL of de-ionized water and transferred to a 18x18 mm square #1.5 glass coverslip (Fisher Scientific, Catalog No. 12541A, 0.15-0.19 mm thickness). The cell suspension was spread over the coverslip surface area as much as possible and left to dry in the dark at room temperature for approximately 30 minutes. *Ex vivo* lung-derived ILC2 were extremely adherent and therefore plated directly onto the glass coverslips in the 6-well plate after the cell sorting process. The washing buffer, fixative solution, and DPBS washes described above were executed at the same concentrations in the 6-well plate by gently aspirating and dispensing the solutions against the edge of the well to minimize detachment of the cells. After the last DPBS wash, these coverslips were left to dry at room temperature in the dark for 30 minutes as well.

Once the coverslips were dry, 70 µL of ProLong™ Gold Antifade Mountant (ThermoFisher Scientific, Catalog No. P36930) was added to the center of each coverslip using low-retention wide-bore pipette tips (Fisher Scientific, Catalog No. 14-222-726). Glass microscopy slides (Fisher Scientific, Catalog No. 12-552-3) were lowered onto the mounting medium until the surface tension pulled the coverslip up and onto the microscope slide to minimize the production of air bubbles. The slides were kept inverted and placed inside a microscope slide box, taking care to not disturb the coverslip. The slides were left in the dark for 24 hours at room temperature to allow the mounting media to cure. Nail polish was traced around the edges of the coverslip to seal and preserve the samples; once the nail polish was dry the samples were stored at 4°C in a closed slide box. All samples were produced in triplicate and imaged within 3 days of the sample preparation.

### 2.14 Image acquisition

Images of ILC2 were acquired as three-dimensional z-stacks on a Zeiss LSM800 AxioObserver Z.1 fully motorized inverted confocal microscope equipped with a 40x/1.30NA Plan Neofluar oil immersion lens. A 405 nm diode laser at 1.0% laser power and 700 gain was tuned to visualize the nucleus (Hoechst 33342) using a multialkali (MA) PMT. A 488 nm diode laser at 0.2% laser power and 700 gain was tuned to visualize lipid droplets (BODIPY 493/503) using a gallium arsenide phosphide (GaAsP) PMT. The following acquisition parameters were used: sequential scanning, 1 Airy unit, 1 digital gain, 1.03 μs pixel dwell time, 4-line averaging, at scan zoom 10. Images were acquired as 1024x1024 pixel frames, with a pixel size of 0.016 x 0.016 μm, and a z-step of 0.5 µm. An average of 10 to 20 frames (5-10 µm depth) were acquired per cell and all image files were saved as 16-bit CZI images (format.czi). An average of 30 cells were acquired per experimental condition, and the same acquisition parameters were used for all conditions.

### 2.15 Image processing

Image files in.czi format were converted to.ims files using the software ImarisFileConverter (Bitplane, Version 10.0.0, Oxford Instruments). The converted files in.ims format were opened in Imaris 3D/4D Visualization and Analysis Software (Bitplane, Version 10.1.0, Oxford Instruments) for analysis of neutral lipid droplets. The *Section* icon was selected to visualize the xy, yz, and xz planes of the image file and an image plane was selected in the center of the cell. A fluorescence intensity line profile was drawn to determine the value of non-specific cytosolic signal in the green channel (BODIPY 493/503 signal). The *Image Proc* icon was then selected for image processing, and the *Baseline Subtraction* function in the green channel (BODIPY 493/503 signal) was selected. The *Baseline Subtraction* threshold was set to the fluorescence intensity previously determined in the *Section* icon and removed from the image to improve the signal-to-background ratio and facilitate neutral LD detection. The same threshold was used for all images within the same parameters.

### 2.16 Neutral lipid droplet detection

Neutral lipid droplet (LD) detection began in the *3D View* and *Surpass* windows of Imaris, where the image file could be viewed as a fully rendered 3D image. Neutral LDs were detected using the *Surface* function, whereby a 3D object was constructed to represent the fluorescent signal in the image file based on a K-means clustering threshold algorithm. The *Surface* function was selected to open the *Creation Wizard* and the option *Start creation with slicer view* was checked before continuing to the next step. Next, the green channel (BODIPY 493/503 signal) was selected under *Source Channel* and the thresholding was set to *Absolute Intensity* before continuing to the next step. In the *Thresholding* step, the thresholding for detecting the fluorescent signal from the neutral LDs was determined. The image file was viewed alternately between the *3D Volume View* and the *Slicer View* while the threshold was being selected to determine the most accurate thresholding value to represent the neutral LD fluorescent signal. When the threshold value was determined to be an accurate representation, the analysis continued to the next step. In the *Filter Surface* step, the *+Add* button was selected under *Filters* to add a *Volume* filter; this filter allowed for the removal of any anomalies based on volume from the overall selection before finalizing the surface construction. The parameters were saved under *Favorite Creation Parameters* so that the same parameters could be used for all image files. Due to the fluorescence intensity variations between large and small neutral LDs, 2-3 different thresholds settings were necessary in most files to detect all the neutral LD and avoid the over- and under-selecting data issue that occurs with using a single threshold. A minimum of 30 cells were analyzed per experimental condition.

### 2.17 Cell volume analysis

Cell volume detection began in the *3D View* and *Surpass* windows of Imaris on.ims formatted files without conducting any image processing. The *Surface* function was selected to open the *Creation Wizard*, and the option *Start creation with slicer view* was checked before continuing to the next step. Next, the green channel (BODIPY 493/503 signal) was selected under *Source Channel* so that cytosolic and auto-fluorescent signals could be used to detect the cell volume. The *Surfaces Detail* was modified to be double the calculated default value (4X the pixel size in total) to improve detection and surface granularity, and the thresholding was set to *Absolute Intensity* before continuing to the next step. In the *Thresholding* step, the thresholding for detecting the cytosolic fluorescent signal was determined. The image file was viewed alternately between the *3D Volume View* and the *Slicer View* while the threshold was being selected to determine the most accurate thresholding value to represent the volume of the cell. When the threshold value was determined to be an accurate representation, the analysis continued to the next step. In the *Filter Surface* step, the *+Add* button was selected under *Filters* to add a *Volume* filter; this filter allowed for the removal of any anomalies based on volume from the overall selection before finalizing the surface construction. The parameters were saved under *Favorite Creation Parameters* so that the same parameters could be used for all image files. A minimum of 30 cells were analyzed per experimental condition.

### 2.18 Data export and quality control

For LD analysis, the *Data Settings* icon was selected in Imaris where the metrics *Intensity Sum, Voxels*, and *Sphericity* were selected under the *Surfaces* category. The *Intensity Sum* is the cumulative fluorescence value of every voxel within the neutral LD surface, *Voxel* is the total number of volumetric pixels in the neutral LD surface, and the *Sphericity* measures how close to a perfect sphere (value of 1.0) the neutral LD surface is. These metrics were exported for every image file in Excel and the data from the green channel (BODIPY 493/503 signal) was consolidated into a master Excel file. Each data point was representative of a single neutral LD, and these datapoints were used to calculate the total neutral lipid fluorescence intensity (*Intensity Sum*) and the total neutral lipid volume (*Voxels*) for each individual cell. Datapoints were removed from the dataset if the sphericity values were above or equal to 1.0 and any voxel values below 10 were considered insignificant. Any datapoints that were abnormally large compared to the distribution of the dataset were reviewed in Imaris for validity before they were removed from the dataset. After the dataset was quality controlled in the manner described above, a MATLAB (Version R2023b, MathWorks, Natick, MA) script was implemented to recalculate the number of neutral LD per cell and to bin the LD into volume (small, intermediate, large) and sphericity (irregular, intermediate, spherical) categories. For cell volume analysis, the *Data Settings* icon was selected in Imaris where the *Voxels* metric selected under the *Surfaces* category. This metric was exported for every image file in Excel and the data from the green channel (BODIPY 493/503 signal) was consolidated into a master Excel file. Each data point was representative of a single cell.

### 2.19 Microscopy graphs and statistical analysis

All data was acquired from three independent experiments for a total of three replicates for each condition. A sample size of 30 cells was chosen to represent each experimental condition to account for normal distribution. All statistical analysis and bar graphs were conducted in the Prism software (Graphpad, Version 9). Statistical analysis for *in vitro* experiments were performed as ordinary one-way ANOVA and *post-hoc* Tukey’s multiple comparison tests to obtain statistical significance (P-values) between experimental conditions. Unpaired two-tailed Student’s t-tests were performed for *ex vivo* experiments to obtain statistical significance (P-values) between populations. All data representing individual cells is presented as mean ± standard deviation (SD), and all data representing individual neutral LD is presented as mean ± standard error of the mean (SEM). Correlation analysis was conducted using JMP 16.1.0 (Statistical Discovery, SAS Institute, NC) to obtain the correlation coefficients between each combination of metrics.

## 3 Results

### 3.1 Activation of group 2 innate lymphoid cells induces fatty acid uptake

As lipid metabolism has been shown to be a significant driver of pathogenic ILC2 function in allergic airway inflammation (33, 39) we aimed at establishing an integrated analytical framework to determine levels of cellular fatty acid (FA) uptake, neutral lipid storage, and fatty acid oxidation (FAO) in primary ILC2 (**Figure 1**). To this end, we obtained primary murine sort purified bone marrow-derived (**Supplementary Figure 1A**) or lung-derived ILC2 (**Supplementary Figure 1B**) that were further expanded *in vitro* as previously described (40, 41). Alternatively, lung-derived ILC2 were obtained by flow cytometric cell sorting (**Supplementary Figure 1B**) from animals that were challenged intranasally with PBS as a control or IL-33 for three consecutive days, which were then further used for *ex vivo* experiments. These three distinct primary murine ILC2 populations were used for the quantification of FA uptake, neutral lipid storage, and FAO by flow cytometry *in vitro* as well as *ex vivo* (**Figure 2**).

**Figure 3:**
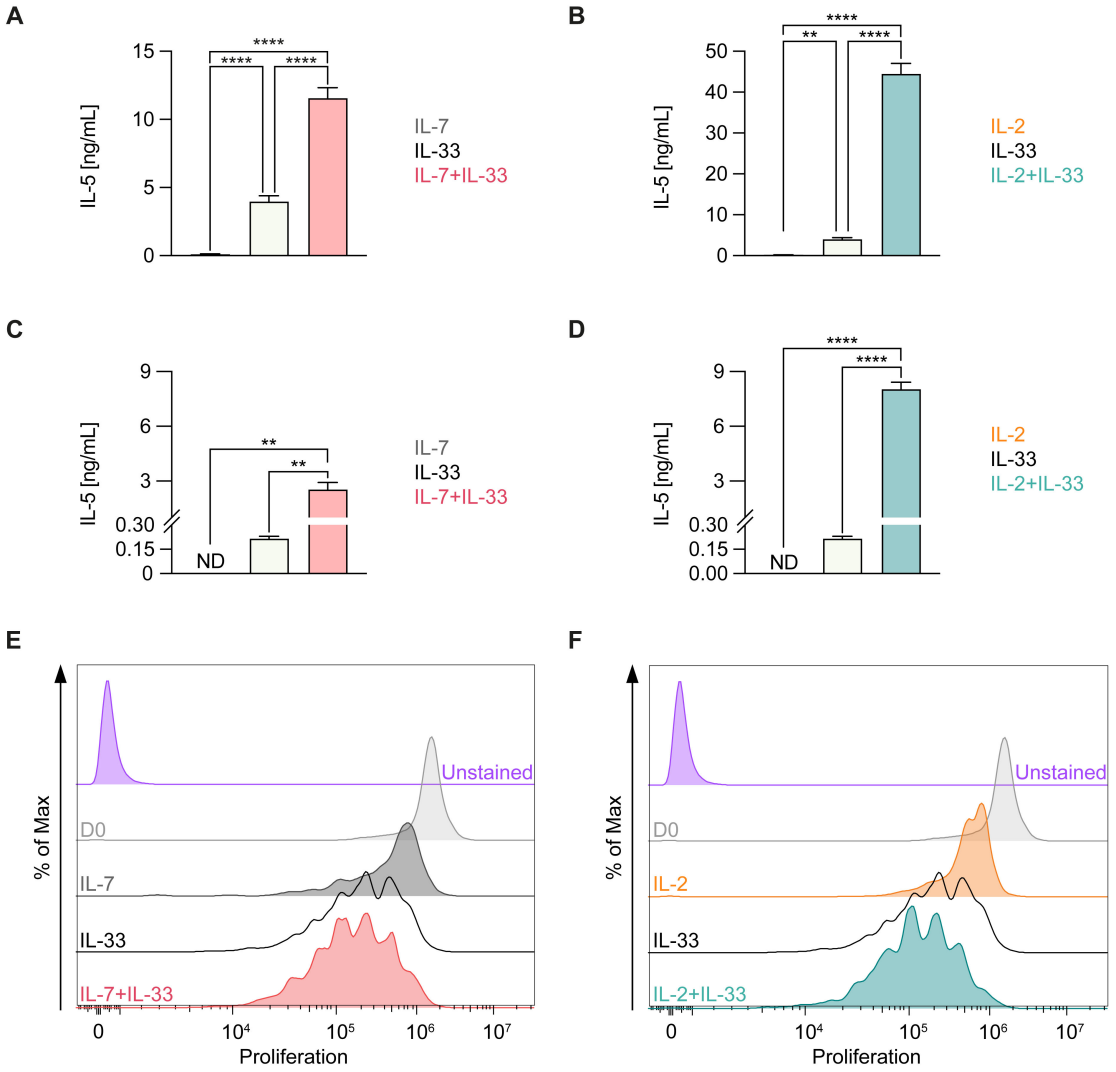
Cytokine production and proliferation of murine ILC2 upon treatment with distinct cytokine combinations. Bone marrow-derived **(A, B, E, F)** or lung-derived **(C, D)** group 2 innate lymphoid cells (ILC2) were stimulated with either IL-7 only, IL-33 only, or a combination of IL-7 and IL-33 **(A, C, E)** or IL-2 only, IL-33 only, or a combination of IL-2 and IL-33 **(B, D, F)**. All cytokines were applied at 10 ng/mL. **(A–D)** After 24 hours of stimulation, supernatants were harvested and analyzed for IL-5 content by ELISA. **(E, F)** Proliferation of ILC2 was assessed using CellTrace Yellow Cell Proliferation Kit after 3 days of incubation with respective cytokines. The data representing the IL-33 stimulation is the same for **(A–F)**. Data are representative of three independent experiments with stimulations performed in duplicates. Data are shown as average ± standard deviation (SD). Statistical analysis was performed using one-way ANOVA followed by Tukey’s multiple comparisons test (p < 0.01 = ** and p < 0.0001 = ****); ND, not detectable.

ILC2 activation by IL-33 has been established as a hallmark of allergic airway inflammation (7). IL-7 and IL-2 secreted by other resident non-hematopoietic stromal cells as well as innate and adaptive immune cells, respectively, have been shown to act in synergy with IL-33 for enhanced ILC2 proliferation and elevated cytokine production (41–43). To first demonstrate the functionality of our ILC2 *in vitro* culture systems, we stimulated bone marrow- (**Figures 3A, B, E, F**) or lung-derived ILC2 (**Figures 3C, D**) with IL-7 (**Figures 3A, C, E**) or IL-2 (**Figures 3B, D, F**) alone or in combination with IL-33 and analyzed the production of IL-5 in cell culture supernatants as well as the proliferative capacity. As previously reported (41), IL-7 and IL-2 act in synergy with IL-33 to induce cell proliferation as well as secretion of type 2 cytokines in both bone marrow- as well as lung-derived ILC2 (**Figures 3A–F**).

**Figure 4:**
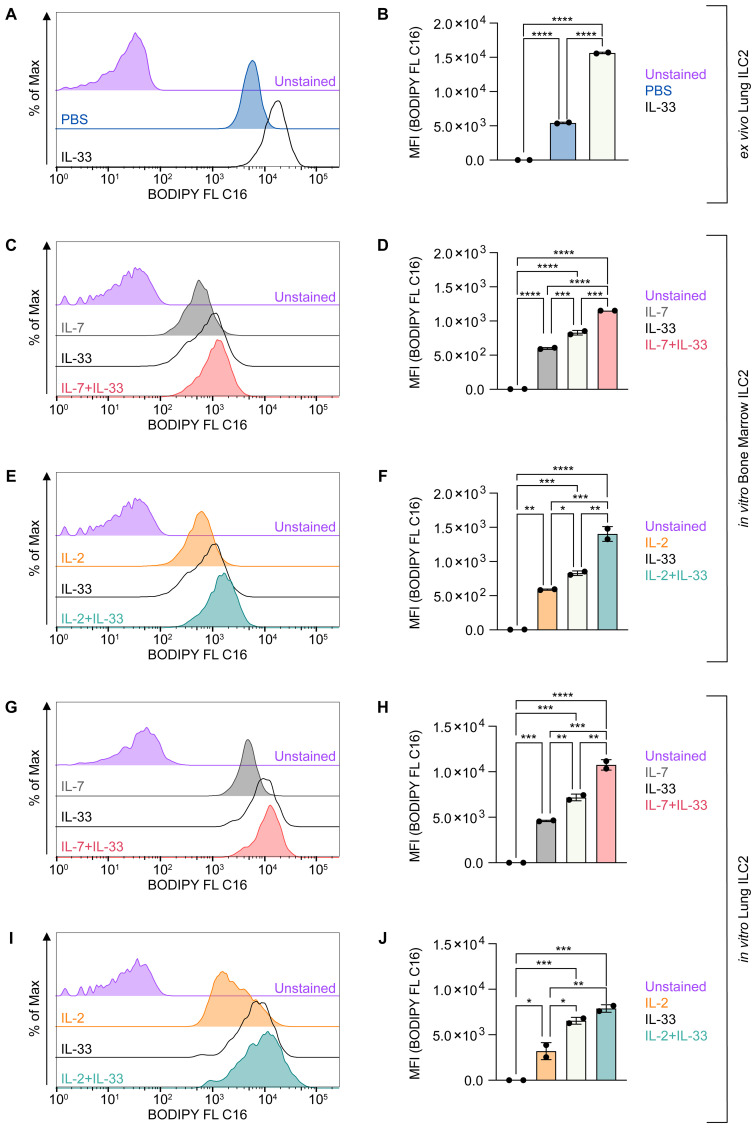
ILC2 increase fatty acid uptake upon stimulation with activating cytokines. **(A, B)** Mice were treated intranasally with either PBS as control or with 250 ng of IL-33 for three consecutive days. Two days after the last treatment, lungs were collected and group 2 innate lymphoid cells (ILC2) were sort-purified and subsequently incubated with BODIPY FL C16 for one hour to assess capacity of lipid uptake by flow cytometric analysis (MFI, geometric mean fluorescence intensity). Bone marrow-derived **(C–F)** or lung-derived **(G–J)** ILC2 were stimulated with either IL-7 only, IL-33 only, or a combination of IL-7 and IL-33 **(C, D, G, H)** or IL-2 only, IL-33 only, or a combination of IL-2 and IL-33 **(E, F, I, J)**. The data representing the IL-33 stimulation is the same for **(C–J)**. All cytokines were applied at 10 ng/mL. BODIPY FL C16 was added after 23 hours of cytokine stimulation and incubated for one hour to assess capacity of lipid uptake by flow cytometric analysis. ILC2 that were not incubated with BODIPY FL C16 served as negative control (Unstained). Data are shown as average ± standard deviation (SD). Statistical analysis was performed using one-way ANOVA followed by Tukey’s multiple comparisons test (p < 0.05 = *, p < 0.01 = **, p < 0.001 = ***, p < 0.0001 = ****).

However, the functions and effects of these cytokines (IL-2, IL-7, IL-33) alone or in synergy (IL-7+IL-33 or IL-2+IL-33) as they pertain to lipid metabolism in ILC2 remains incompletely understood. Therefore, to investigate the ability of ILC2 to acquire exogenous FAs during defined activation states, we optimized a flow cytometric assay, by incubating ILC2 *ex vivo* or *in vitro* with BODIPY FL C16, a fluorescent FA analog (**Figure 4**). We first analyzed sort-purified ILC2 from mice that were treated intranasally with PBS or IL-33 *ex vivo* (**Figures 4A, B**). Although ILC2 from PBS-treated mice acquired exogenous FAs, ILC2 from IL-33 treated animals had a significantly higher FA intake (**Figures 4A, B**). To further analyze how distinct activating cytokines impact FA uptake by ILC2, bone marrow-derived (**Figures 4C–F**) and lung-derived ILC2 (**Figures 4G–J**) were treated *in vitro* with IL-7, IL-2, IL-33 alone or with combinations of IL-7+IL-33 or IL-2+IL-33 for 24 hours. BODIPY FL C16 was then added 23 hours after cytokine stimulation, incubated for one hour and subsequently analyzed by flow cytometry (**Figures 4C–J**). Similarly to ILC2 analyzed *ex vivo* (**Figures 4A, B**), bone-marrow and lung-derived ILC2 treated with IL-7 or IL-2 alone display moderate exogenous FA intake, whereas IL-33 treated ILC2 exert a significant increase in FA acquisition (**Figures 4C–J**). Furthermore, bone marrow-derived ILC2 treated with the combination of IL-7+IL-33 or IL-2+IL-33 significantly increased FA uptake when compared to IL-7, IL-2 or IL-33 alone (**Figures 4C–F**). Lung-derived ILC2 were also found to acquire more exogenous FAs when stimulated by IL-7+IL-33 compared to IL-7, or IL-33 alone, but no significant increase was observed comparing stimulations with IL-33 alone vs IL-2+IL-33 (**Figures 4G–J**). Collectively, these observations demonstrate that ILC2 are actively taking up FA from the environment at steady state, which is further reinforced by stimulation with IL-33 only as well as by synergistic activation with IL-7+IL-33 or IL-2+IL-33.

### 3.2 Activation of group 2 innate lymphoid cells induces increased storage of neutral lipids

**Figure 5:**
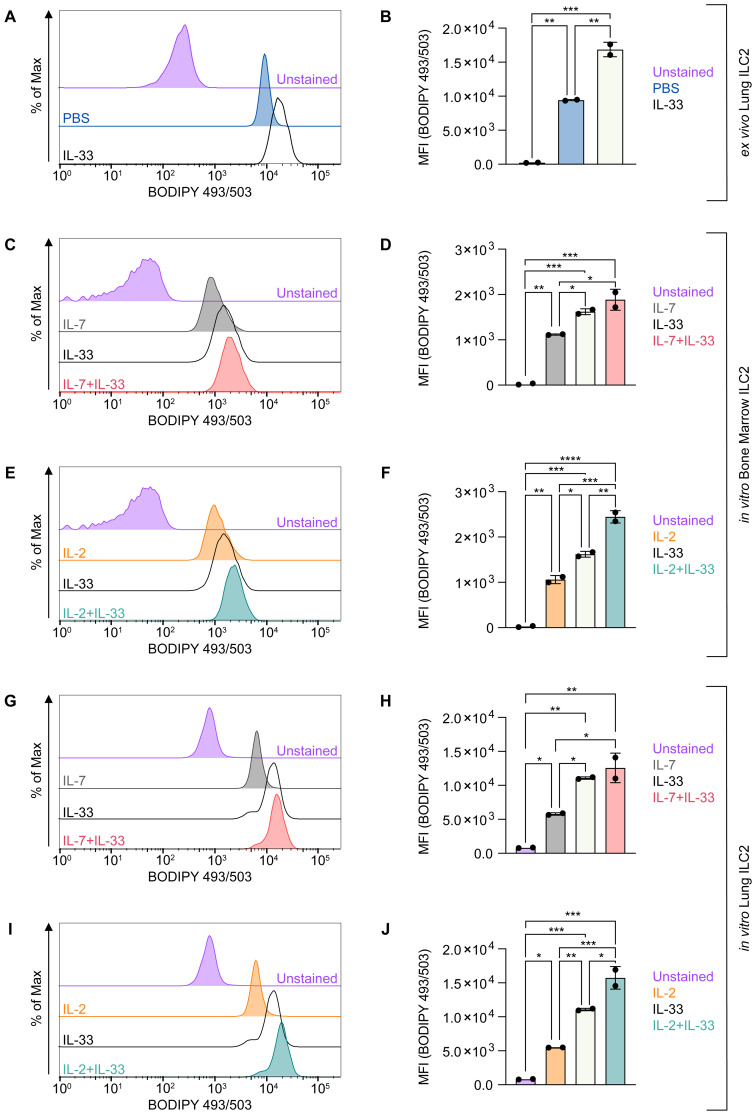
ILC2 increase neutral lipid storage upon stimulation with activating cytokines. **(A, B)** Mice were treated intranasally with either PBS as control or with 250 ng of IL-33 for three consecutive days. Two days after the last treatment, lungs were collected and group 2 innate lymphoid cells (ILC2) were sorted and subsequently incubated with BODIPY 493/503 for 20 minutes to assess capacity of neutral lipid storage by flow cytometric analysis (MFI, geometric mean fluorescence intensity). Bone marrow-derived **(C–F)** or lung-derived **(G–J)** ILC2 were stimulated with either IL-7 only, IL-33 only, or a combination of IL-7 and IL-33 **(C, D, G, H)** or IL-2 only, IL-33 only, or a combination of IL-2 and IL-33 **(E, F, I, J)**. The data representing the IL-33 stimulation is the same for **(C–J)**. All cytokines were applied at 10 ng/mL. BODIPY 493/503 was added after 24 hours of cytokine stimulation and incubated for 20 minutes to assess capacity of neutral lipid storage by flow cytometric analysis. ILC2 that were not incubated with BODIPY 493/503 served as negative control (Unstained). Data are shown as average ± standard deviation (SD). Statistical analysis was performed using one-way ANOVA followed by Tukey’s multiple comparisons test (p < 0.05 = *, p < 0.01 = **, p < 0.001 = ***, p < 0.0001 = ****).

To further analyze to which extent FA uptake correlates with storage of neutral lipids we optimized a flow-cytometry-based protocol, enabling the rapid quantification of neutral lipid storage by ILC2. First, lung ILC2 were sort-purified from mice challenged intranasally with PBS as control or IL-33 for three consecutive days and were then stained *ex vivo* with BODIPY 493/503 for 20 minutes at 37 degrees Celsius. At steady state (PBS control treatment), ILC2 were found to store moderate levels of neutral lipids, while upon IL-33 treatment ILC2 considerably increased their neutral lipid storage (**Figures 5A, B**). To understand how cytokines known to drive ILC2 effector functions regulate neutral lipid storage, bone marrow- or lung-derived ILC2 were incubated with IL-7, IL-2 and IL-33 alone, or with combinations of IL-7+IL-33 or IL-2+IL-33 for 24 hours and subsequently stained with BODIPY493/503 (**Figures 5C–J**). Neutral lipid storage of bone marrow-derived ILC2 (**Figures 5C–F**) or lung-derived ILC2 (**Figures 5G–J**) treated with IL-7 or IL-2 alone was low but considerably increased in the presence of IL-33 (**Figures 5C–J**). Furthermore, combined cytokine treatment with IL-2+IL-33 significantly increased the accumulation of neutral lipids compared to stimulations with IL-2 or IL-33 alone in bone marrow-derived ILC2 (**Figures 5E, F**) as well as lung-derived ILC2 (**Figures 5I, J**). In contrast, no significant elevation of neutral lipid storage was observed in bone marrow-derived ILC2 (**Figures 5C, D**) as well as lung-derived ILC2 (**Figures 5G, H**) when comparing treatments of IL-33 alone vs IL-7+IL-33. However, the combined cytokine treatment of IL-7+IL-33 showed significantly higher levels of neutral lipid storage when compared to IL-7 alone (**Figure 5C, D, G, H**). These findings show that ILC2 at steady state harbor moderate levels of neutral lipids, actively increase their neutral lipid content upon treatment with IL-33, but especially when synergistically activated by IL-2+IL-33.

### 3.3 Activation of group 2 innate lymphoid cells induces mitochondrial β-oxidation of fatty acids

Studies investigating the impact of ILC2-specific deletion of DGAT1 (33) and PPARγ (33–35, 44), genes involved in lipid droplet formation (45) and lipid metabolism (46), respectively, as well as insights gained by pharmacological inhibition of Cpt1a through Etomoxir in *in vitro* and *in vivo* assays (32, 39) led to the conclusion that lipid metabolism constitutes a significant driver of pathogenic ILC2 function in allergic airway inflammation. These insights were further substantiated by the observations that PPARγ deletion impacts mitochondrial activities of ILC2 (35) and that FA supplementation of ILC2 cultures increases oxygen consumption rates (OCR) (33). However, it remains poorly defined to which extent FAs taken up from the extracellular environment or catabolized from stored triglyceride (TG) pools serve as a critical energy source and mechanistic driver for ILC2 proliferation and type 2 cytokine production. We therefore developed a rapid and easily applicable flow cytometry-based assay that enables direct analysis of mitochondrial β-oxidation levels of FAs in living ILC2 *ex vivo* and *in vitro* utilizing a recently developed reagent referred to as FAOBlue (47). FAOBlue is a coumarin dye coupled to a nonanoic acid (C9), which is protected by an acetoxymethyl ester, showing no fluorescence at 405 nm. FAOBlue can simply enter cells as it is permeable to the cell membrane. Upon cytosolic localization the acetoxymethyl ester is hydrolyzed by intracellular esterases, providing the free FA type of FAOBlue, which is further converted to an acyl-CoA form and thereby incorporated into the FAO pathway. Subsequently, acyl-CoA-type FAOBlue is then degraded by three FAO cycles to non-fluorescent coumarin possessing a propionic acid (C3). Then after the 4^th^ FAO cycle degradation, the coumarin dye is released from the propionic acid. The released coumarin dye derived from FAO cycles exerts strong blue fluorescence excited by 405 nm, allowing sensitive detection of FAO activity in living cells.

**Figure 6:**
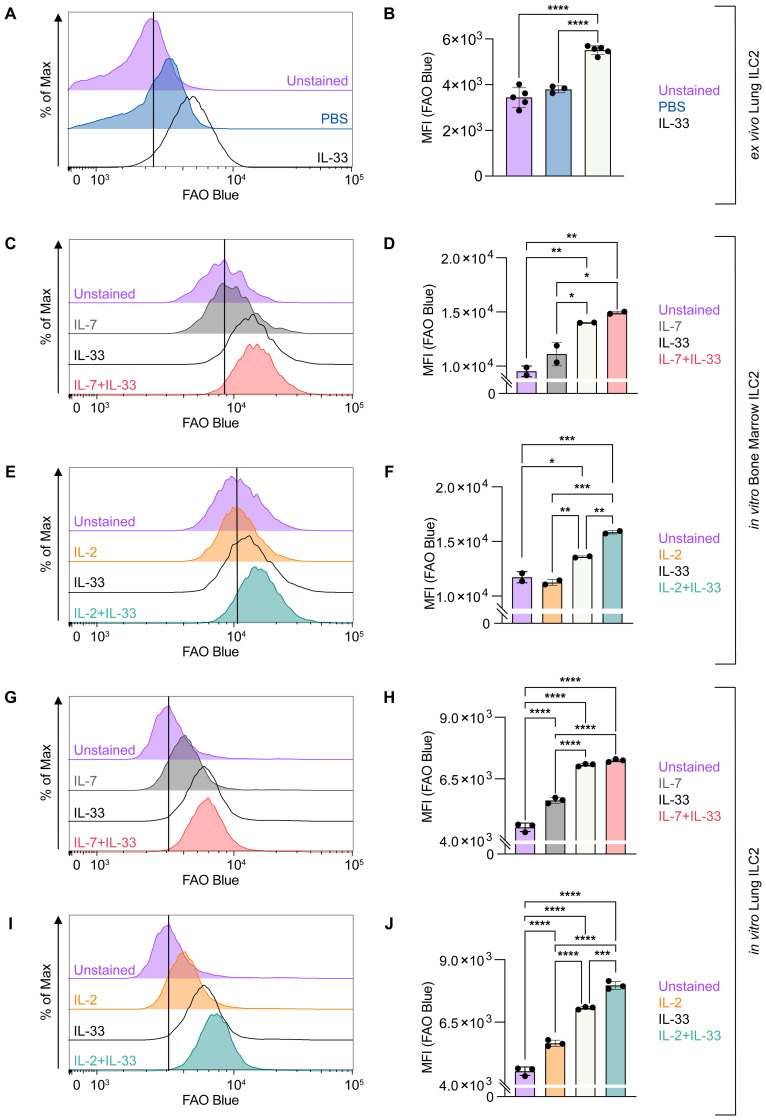
ILC2 increase fatty acid oxidation upon stimulation with activating cytokines. **(A, B)** Mice were treated intranasally with either PBS as control or with 250 ng of IL-33 for three consecutive days. Two days after the last treatment, lungs were collected, and group 2 innate lymphoid cells (ILC2) were sorted and subsequently stained with FAO Blue for one hour to assess capacity of fatty acid oxidation by flow cytometric analysis (MFI, geometric mean fluorescence intensity). Bone marrow-derived **(C–F)** or lung-derived **(G–J)** ILC2 were stimulated with either IL-7 only, IL-33 only, or a combination of IL-7 and IL-33 **(C, D, G, H)** or IL-2 only, IL-33 only, or a combination of IL-2 and IL-33 **(E, F, I, J)**. The data representing the IL-33 stimulation is the same for **(C–J)**. All cytokines were applied at 10 ng/mL. After 24 hours of cytokine stimulation cells were incubated for one hour with FAO Blue and analyzed using flow cytometry. ILC2 that were not incubated with FAO Blue served as negative control (Unstained). Data are shown as average ± standard deviation (SD). Statistical analysis was performed using one-way ANOVA followed by Tukey’s multiple comparisons test (p < 0.05 = *, p < 0.01 = **, p < 0.001 = ***, p < 0.0001 = ****).

We first applied FAOBlue to assess the extent by which ILC2 use mitochondrial FAO at steady state compared to IL-33-induced airway inflammation *in vivo* (**Figures 6A, B**). To this end mice were challenged intranasally with PBS or IL-33 for three consecutive days. Pulmonary ILC2 were sort-purified 48 hours after the last treatment and stained *ex vivo* with FAOBlue dye for 1 hour at 37 degrees Celsius (**Figures 6A, B**). At steady state, pulmonary ILC2 displayed moderate levels of FAO while intranasal IL-33 treatment led to a significant increase of FAO by ILC2 (**Figures 6A, B**). To investigate how cytokines known to drive ILC2 effector functions regulate FAO, bone marrow- or lung-derived ILC2 were incubated with IL-7, IL-2 and IL-33 alone or with combinations of IL-7+IL-33 or IL-2+IL-33 for 24 hours and were subsequently stained with FAOBlue (**Figures 6C–J**). FAO by bone marrow-derived ILC2 (**Figures 6C–F**) or lung-derived ILC2 (**Figures 6G–J**) treated with IL-7 or IL-2 alone was moderate but considerably increased in the presence of IL-33 (**Figures 6C–J**). Furthermore, combined cytokine treatment with IL-2+IL-33 significantly increased FAO compared to stimulations with IL-2 or IL-33 alone in bone marrow-derived ILC2 (**Figures 6E, F**) as well as lung-derived ILC2 (**Figures 6I, J**). In contrast, no significant elevation of FAO was observed in bone marrow-derived ILC2 (**Figures 6C, D**) as well as lung-derived ILC2 (**Figures 6G, H**) when comparing treatments of IL-33 alone vs IL-7+IL-33. However, the combined cytokine treatment of IL-7+IL-33 showed significantly higher levels of FAO when compared to IL-7 alone (**Figure 6C, D, G, H**). These findings demonstrate that FAO by ILC2 is low, significantly increases by stimulation with IL-33, but were found to be highest when ILC2 were synergistically activated by IL-2+IL-33.

### 3.4 Cytokine stimulation of group 2 innate lymphoid cells induces specific morphologies of neutral lipid droplets

ILC2 are a rare, non-adherent type of innate immune cell, making them an excellent candidate for flow cytometric assays and analysis. However, these two factors make ILC2 samples difficult to prepare for microscopy and therefore visualization of intracellular morphologies of ILC2 has been largely unexplored. The majority of ILC2 images that exist are from the ImageStream platform where a fluorescent 2D projection of a 3-dimensional cell is used primarily for qualitative purposes (33, 40). Furthermore, microscopy sample preparation of non-adherent cells traditionally implements either the use of a cytospin which risks bursting the cell membrane or disrupting intracellular architecture (48), or extracellular matrix coverslip coatings that influence protein expression at the cell membrane to promote cell adhesion (49). In Section 3.2, using flow cytometry, we demonstrated that activation of ILC2 induces increased storage of neutral lipids, but these assays did not provide detailed insights of these intracellular lipid pools. To elucidate the morphologies and distribution of neutral lipids in ILC2, we developed a novel sample preparation protocol for acquiring high-resolution 3D images for lipid droplet quantification that conserves ILC2 morphology across equipment platforms.

**Figure 7:**
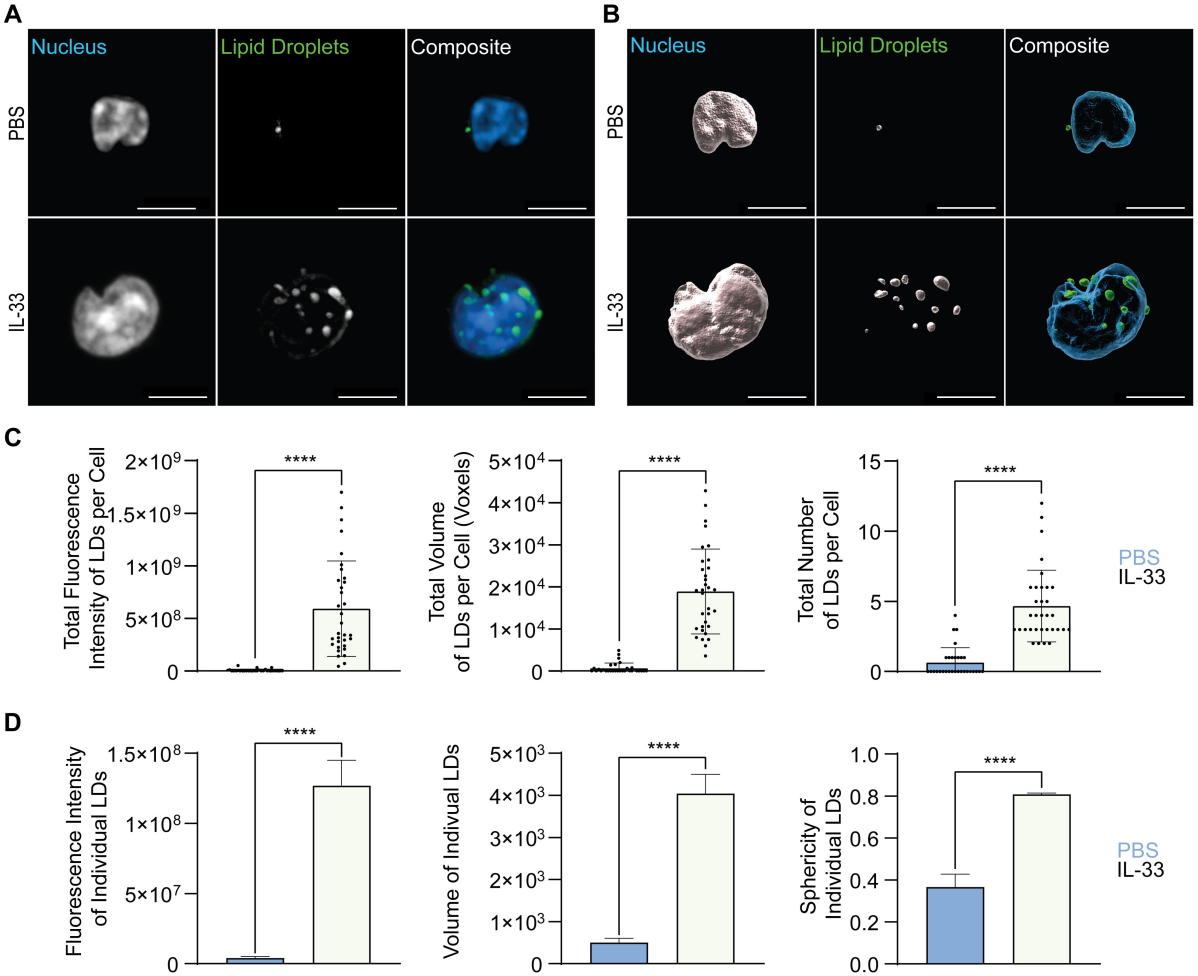
Allergic airway inflammation increases the lipid droplet number and size in pulmonary ILC2. Mice were treated intranasally with either PBS as control or with 250 ng of IL-33 for three consecutive days. Two days after the last treatment, lungs were collected, and group 2 innate lymphoid cells (ILC2) were sorted by flow cytometry and labelled with Hoechst 33342 to visualize the nucleus (blue) and BODIPY 493/503 to visualize neutral lipid storage **(A, B)**. Confocal images **(A)** and 3D fluorescence reconstructions from Imaris **(B)** are shown (PBS - top row; IL-33 treatments - bottom row). Confocal images are displayed as a maximum intensity projection of approximately 10-20 frames (5-10 μm depth) produced in ImageJ. Images are representative from 3 independent experiments, scale bars represent 5 μm. **(C)** Quantification of the total fluorescence intensity (left), total volume (middle), and total number (right) of neutral lipid droplets cumulatively per cell. Fluorescence and morphology metrics were taken from individual neutral lipid droplets and were added together based on cell of origin and then by intranasal challenge. ILC2 that underwent PBS intranasal challenge are shown in blue (n = 30) and those treated with IL-33 are shown in off-white (n = 33). **(D)** Quantification of the total fluorescence intensity (left), total volume (middle), and sphericity (right) of individual neutral lipid droplets. Fluorescence and morphology metrics were taken from the same individual neutral lipid droplets shown in **(C)** and compiled based on intranasal challenge alone; PBS (n = 39) and IL-33 (n = 154). The data representing the IL-33 stimulation is the same for A-D. Data are represented as average ± standard deviation (SD) where n = number of cells **(C)** or average ± standard error of measurement (SEM) where n = number of neutral lipid droplets **(D)**. Statistical significance (*p*-values) was calculated using unpaired two-tailed Student’s t-test (p < 0.0001 = ****).

First, lung ILC2 were sort-purified from mice challenged intranasally with PBS as control or IL-33 for three consecutive days and were then stained *ex vivo* with BODIPY 493/503 for 30 minutes at 37˚C prior to fixation, mounting, and imaging (**Figure 7A**). These microscopy images were analyzed in software that allowed for 3D reconstruction of the neutral lipid fluorescent signal from which fluorescence intensity and morphology metrics could be extracted (**Figure 7B**). At steady state (PBS control treatment), ILC2 were shown to store low total fluorescence intensity of neutral lipids concurrent with the flow cytometry data (**Figure 5B**), explained by a low total volume and number of lipid droplets (LDs) per cell (**Figure 7C**). When treated with IL-33, the total neutral lipid fluorescence intensity, total volume, and number of LDs per cell dramatically increased (**Figure 7C**).

**Figure 8:**
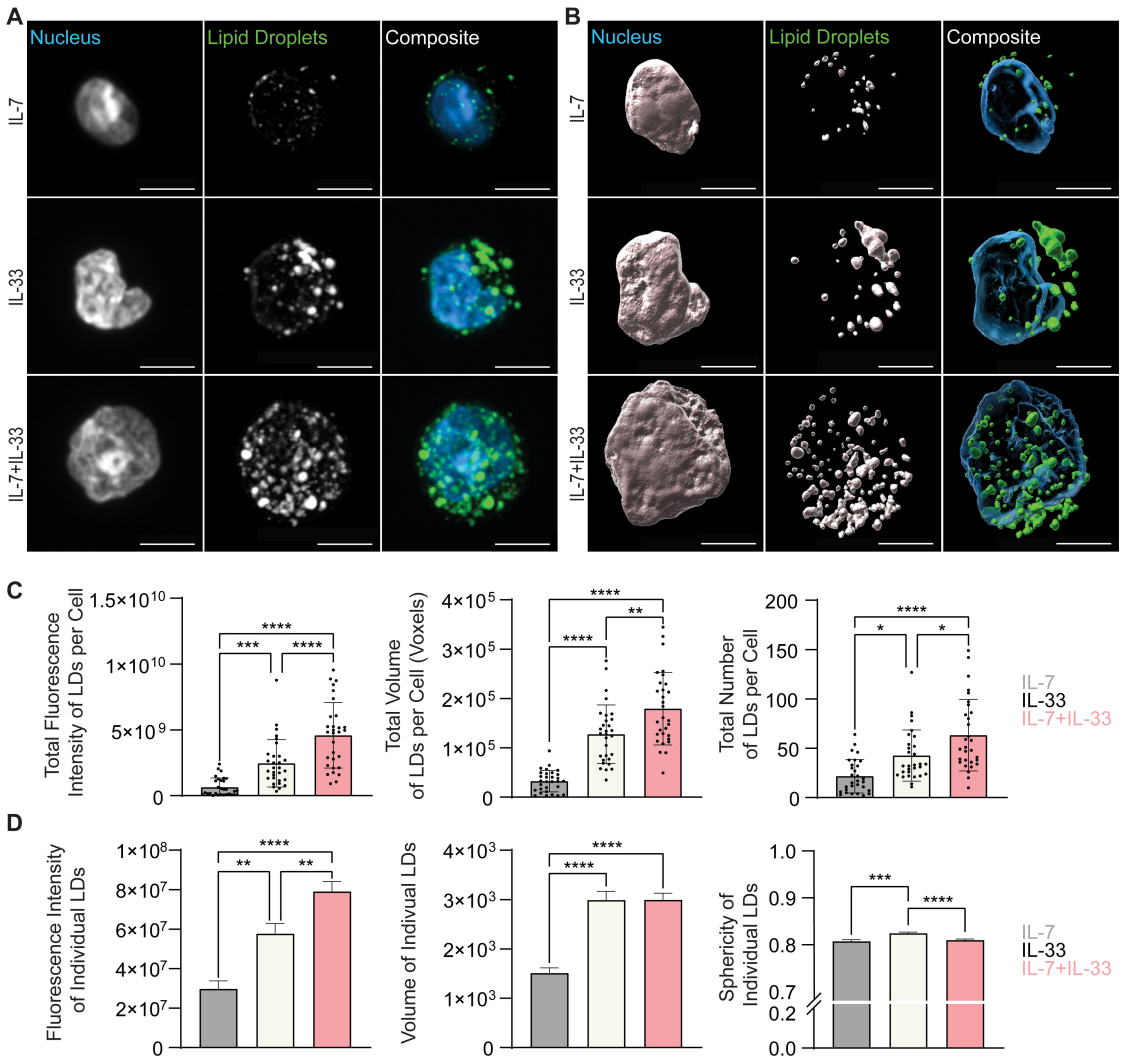
*In vitro* stimulation of ILC2 with combinations of IL-7 and IL-33 changes the neutral lipid droplet size and number. Bone marrow-derived group 2 innate lymphoid cells (ILC2) were stimulated for 24 hours with either IL-7 only, IL-33 only, or a combination of IL-7 and IL-33 (all cytokines were applied at 10 ng/mL) and labelled with Hoechst 33342 to visualize the nucleus (blue) and BODIPY 493/503 to visualize neutral lipid storage **(A, B)**. Confocal images **(A)** and 3D fluorescence reconstructions from Imaris **(B)** are shown. Confocal images are displayed as a maximum intensity projection of approximately 10-20 frames (5-10 μm depth) produced in ImageJ. Images are representative from 3 independent experiments, scale bars represent 5 μm. **(C)** Quantification of the total fluorescence intensity (left), total volume (middle), and total number (right) of neutral lipid droplets cumulatively per cell. Fluorescence and morphology metrics were taken from individual neutral lipid droplets and were added together based on cell of origin and then by cytokine treatment. ILC2 were treated with either IL-7 (grey, n = 30), IL-33 (off-white, n = 30), or IL-7+IL-33 (pink, n =30) for 24 hours prior to imaging. **(D)** Quantification of the total fluorescence intensity (left), total volume (middle), and sphericity (right) of individual neutral lipid droplets. Fluorescence and morphology metrics were taken from the same individual neutral lipid droplets shown in **(C)** and were compiled based on cytokine treatment alone; IL-7 (n = 637), IL-33 (n = 1,267), or IL-7+IL-33 (n = 1,569). The data representing the IL-33 stimulation is the same for **(A–D)**. Data are represented as average ± standard deviation (SD) where n = the number of cells **(C)** or average ± standard error of measurement (SEM) where n = number of neutral lipid droplets **(D)**. Statistical significance (*p*-values) was calculated using ordinary one-way ANOVA and *post-hoc* Tukey’s multiple comparison tests (p < 0.05 = *, p < 0.01 = **, p < 0.001 = ***, p < 0.0001 = ****).

**Figure 9:**
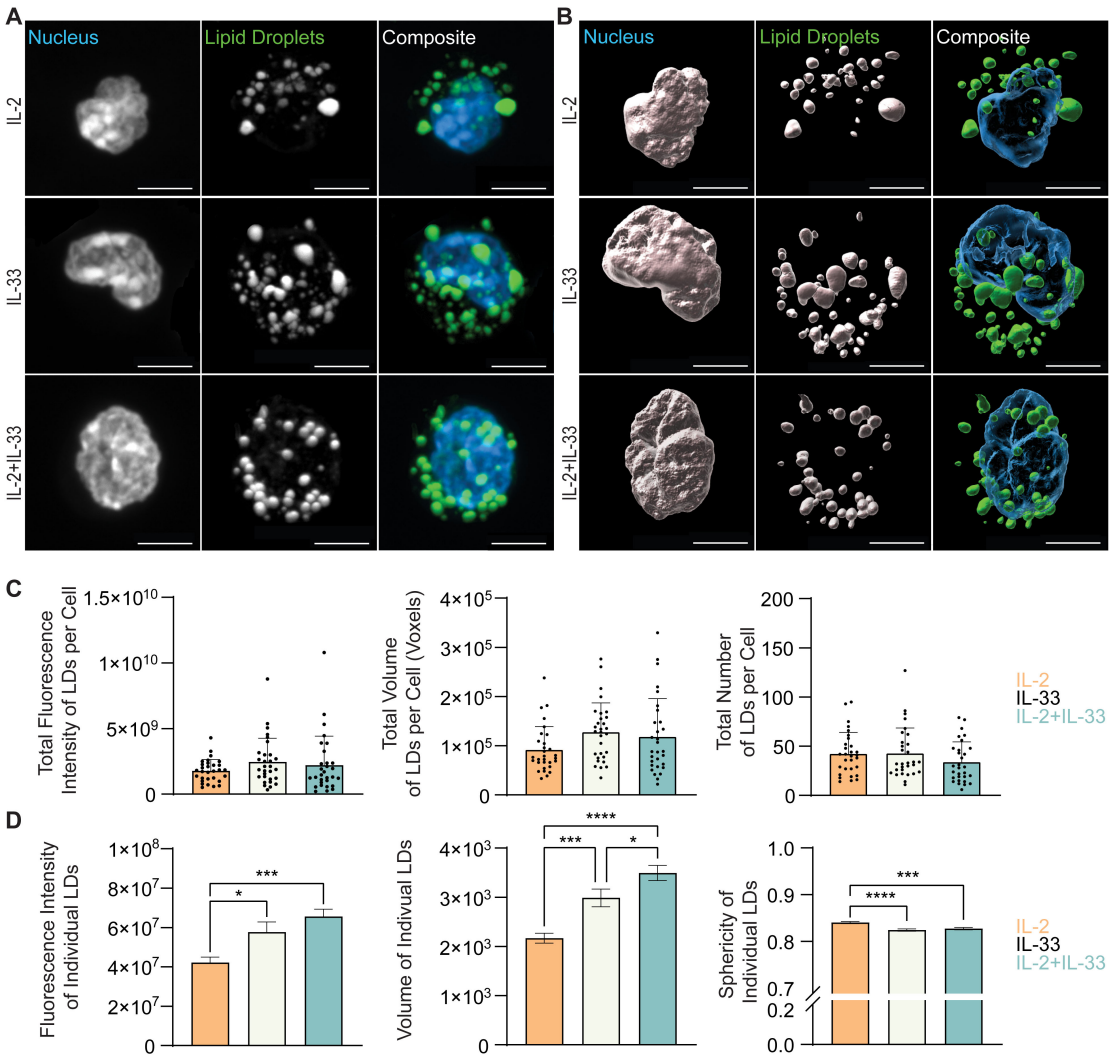
*In vitro* stimulation of ILC2 with combinations of IL-2 and IL-33 changes the neutral lipid droplet volume. Bone marrow-derived group 2 innate lymphoid cells (ILC2) were stimulated for 24 hours with either IL-2 only, IL-33 only, or a combination of IL-2 and IL-33 (all cytokines were applied at 10 ng/mL) and labelled with Hoechst 33342 to visualize the nucleus (blue) and BODIPY 493/503 to visualize neutral lipid storage **(A, B)**. Confocal images **(A)** and 3D fluorescence reconstructions from Imaris **(B)** are shown. Confocal images are displayed as a maximum intensity projection of approximately 10-20 frames (5-10 μm depth) produced in ImageJ. Images are representative from 3 independent experiments, scale bars represent 5 μm. **(C)** Quantification of the total fluorescence intensity (left), total volume (middle), and total number (right) of neutral lipid droplets cumulatively per cell. Fluorescence and morphology metrics were taken from individual neutral lipid droplets and were added together based on cell of origin and then by cytokine treatment. ILC2 were treated with either IL-2 (orange, n = 30), IL-33 (off-white, n = 30), or IL-2+IL-33 (teal, n = 30) for 24 hours prior to imaging. **(D)** Quantification of the total fluorescence intensity (left), total volume (middle), and sphericity (right) of individual neutral lipid droplets. Fluorescence and morphology metrics were taken from the same individual neutral lipid droplets in **(C)** and compiled based on cytokine treatment alone; IL-2 (n = 1,266), IL-33 (n = 1,267), or IL-2+IL-33 (n = 1,015). The data representing the IL-33 stimulation is the same for **(A–D)**. Data are represented as average ± standard deviation (SD) where n = the number of cells **(C)** or average ± standard error of measurement (SEM) where n = number of neutral lipid droplets **(D)**. Statistical significance (*p*-values) was calculated using ordinary one-way ANOVA and *post-hoc* Tukey’s multiple comparison tests (p < 0.05 = *, p < 0.001 = ***, p < 0.0001 = ****).

To further understand how cytokines known to drive ILC2 effector functions regulate neutral lipid storage, *in vitro* bone marrow-derived ILC2 were incubated with IL-7, IL-2, or IL-33 alone, or with combinations of IL-7+IL-33 or IL-2+IL-33 for 24 hours. These cells were subsequently stained with BODIPY493/503, fixed, mounted, and imaged (**Figures 8A**, **9A**) followed by 3D image analysis (**Figures 8B**, **9B**). Similarly to the *ex vivo* lung-derived ILC2, the total fluorescence intensity, total volume, and number of all neutral LDs per cell presented by *in vitro* bone marrow-derived ILC2 treated with IL-7 alone were significantly lower in comparison to all other cytokine treatments (**Figures 8A–C**). Marked elevation in the total fluorescence intensity, total volume, and number of all LDs in the presence of IL-33 alone, as well as with the combined treatment of IL-7+IL-33 was observed (**Figures 8A–C**). In contrast, treatment of ILC2 with IL-2 only already led to high levels of total fluorescence intensity, total volume, and number of all LDs (**Figures 9A–C**). In addition, when compared to stimulations by IL-2 alone, no significant changes in total fluorescence intensity, total volume, or number of all LDs per cell compared to treatments with IL-33, or a combination of IL-2+IL-33 (**Figures 9A–C**) were observed.

Microscopy is a very time-consuming process, easily susceptible to sample heterogeneity, where the target sample number is approximately 30 cells per condition. To overcome these obstacles, we further investigated the morphological neutral LD diversity as an entire population outside the confines of individual cell analysis. Individual neutral LDs in pulmonary ILC2 isolated from naïve mice (PBS control treatments), exhibited a small volume, were irregularly shaped, and low in fluorescence intensity (**Figure 7D**). In contrast, in lung ILC2 isolated from IL-33 treated animals, the individual neutral LDs significantly increased in volume, became more spherical, and subsequently increased in fluorescence intensity (**Figure 7D**).

When analyzing bone marrow-derived ILC2 treated with distinct cytokines *in vitro*, we revealed that the fluorescence intensity and volume of the individual neutral LDs is low in cells treated with IL-7 (**Figure 8D**) or IL-2 alone (**Figure 9D**) but considerably increased when treated with IL-33. Furthermore, ILC2 treated with cytokine combinations IL-7+IL-33 or IL-2+IL-33 markedly increased the fluorescence intensity as well as the volume of individual LDs compared to stimulations with either IL-7 (**Figure 8D**) or IL-2 (**Figure 9D**) alone. While a significant elevation in neutral LD fluorescence intensity was observed in ILC2 treated with IL-7+IL-33 compared to IL-33 only stimulations, no changes with regards to LD volume were found (**Figure 8D**). Moreover, while no significant differences in neutral LD fluorescence intensity were observed in ILC2 stimulated with IL-33 alone versus IL-2+IL-33, a significant elevation in LD volume was observed between the two treatment groups (**Figure 9D**). In addition, the neutral LDs of ILC2 stimulated with IL-33 alone were closer to a perfect sphere compared to treatments with IL-7 alone or IL-7+IL-33 (**Figure 8D**). In contrast, the LDs in ILC2 activated with IL-2 only were the closest to a perfect sphere compared to stimulations with IL-33 alone or IL-2+IL-33 (**Figure 9D**). These findings demonstrate that the cytokines known to drive ILC2 effector functions play a significant role in determining morphology of neutral LDs.

### 3.5 Cytokine stimulation of group 2 innate lymphoid cells induces a shift in the distribution of neutral lipid droplet morphology

When the individual neutral LD datapoints are compiled into a single value to represent an entire cell, potential information is lost. However, additional valuable insight can be uncovered by investigating the distribution of the datapoints within each cell, as opposed to only taking an average of analyzed values. To address this query, we binned the volume and sphericity (**Table 3**) of individual LDs for each cell into categories and took the average of each category’s frequency. The LD volumes were separated into large, intermediate, and small sized droplets, and the sphericity of each lipid droplet was described as either spherical, intermediate, or irregularly shaped.

**Table 3:** Distribution of neutral lipid droplet morphology.

Lipid Droplet Volume					
Bin Size	IL-7	IL-2	IL-33	IL-7+IL-33	IL-2+IL-33
Large (Above 5,000)	10	13	16	19	24
Intermediate (1,000 to 4,999)	33	36	46	48	49
Small (Below 1,000)	57	51	38	33	27
Lipid Droplet Sphericity					
Bin Size	IL-7	IL-2	IL-33	IL-7+IL-33	IL-2+IL-33
Spherical (Above 0.85)	42	59	51	49	56
Intermediate (0.7 to 0.84)	48	36	40	41	37
Irregular (Below 0.7)	10	5	9	10	7

The average distribution (in %) of lipid droplet volumes (top) and sphericity (bottom) per cell between each cytokine stimulation population. Lipid droplet volumes are categorized as large, intermediate, or small. Lipid droplet sphericity is categorized as spherical, intermediate, or irregular-shaped. Each row is color-coded to distinguish shifts in distribution where red indicates the lowest value in the row, yellow is median, and green is the highest value.

When comparing the frequencies of each morphology metric between cytokine stimulations *in vitro*, there is a shift in the morphology of neutral LDs between the different cytokines (**Table 3**). IL-7 holds the lowest average proportion of large neutral LDs compared to other cytokine stimulations at 10% of the population, whereas IL-2+IL-33 has the highest average proportion at 24%. This correlates with the inverse as well, where IL-7 holds the highest average proportion of small neutral LDs at 57% of the population, and IL-2+IL-33 has the smallest average proportion at 27%. There is a marked shift in the distribution of the LD volume between either IL-7 or IL-2 alone and the respective combination treatment with IL-7+IL-33 or IL-2+IL-33 (**Table 3**). The average proportion of large neutral LDs in IL-2 and IL-7 nearly doubles when combined with IL-33, and the average proportion of small neutral LDs specifically in IL-2 nearly halves (**Table 3**). This distribution data sheds light on a trend that the specific combination of IL-7+IL-33 produces both large and small LDs, whereas the IL-2+IL-33 treated LDs shift towards larger and intermediate-sized LDs, and away from smaller LDs as observed in the representative microscopy images (**Figures 8A, B**, **9A, B**). Furthermore, the neutral LDs in IL-7 have the most intermediate and irregularly shaped LDs, whereas the LDs in IL-2 have the most spherical (**Table 3**). Interestingly, the LDs in IL-7 are mostly irregularly or intermediately shaped and shift towards being more spherical upon activation with IL-7+IL-33, whereas LDs in IL-2 alone exhibit a shift to become less spherical with IL-33 activation (**Table 3**). In both LD volume and sphericity, IL-33 is shown to be a true median between the solitary treatments of IL-7 and IL-2 and the activation of ILC2 with the combinations of IL-7+IL-33 and IL-2+IL-33 (**Table 3**). The data acquired from the *ex vivo* dataset was not included in the distribution analysis due to the infrequent presence of neutral LDs in the PBS-treated ILC2. Taken together, these data demonstrate that each cytokine induces specific phenotypes in neutral LD storage that may be related to the catabolism and anabolism of LDs in ILC2.

### 3.6 Activation of group 2 innate lymphoid cells increases the cell volume

Cell volume can be estimated using flow cytometry by investigating forward scatter (FSC) and side scatter (SSC) values, however these metrics are merely comparative without the application of beads with a known size. Fluorescence microscopy offers an advantage where cytosolic fluorescence or autofluorescence can be utilized to calculate the volume of a cell. Previous studies have qualitatively observed that ILC2 increase in cell volume following activation, but this has not been extensively quantified (50). Increases in cell volume require the synthesis of phospholipids for cell membrane production, which have been traced from exogenous FAs transiently stored in neutral LDs in ILC2 (33).

**Figure 10:**
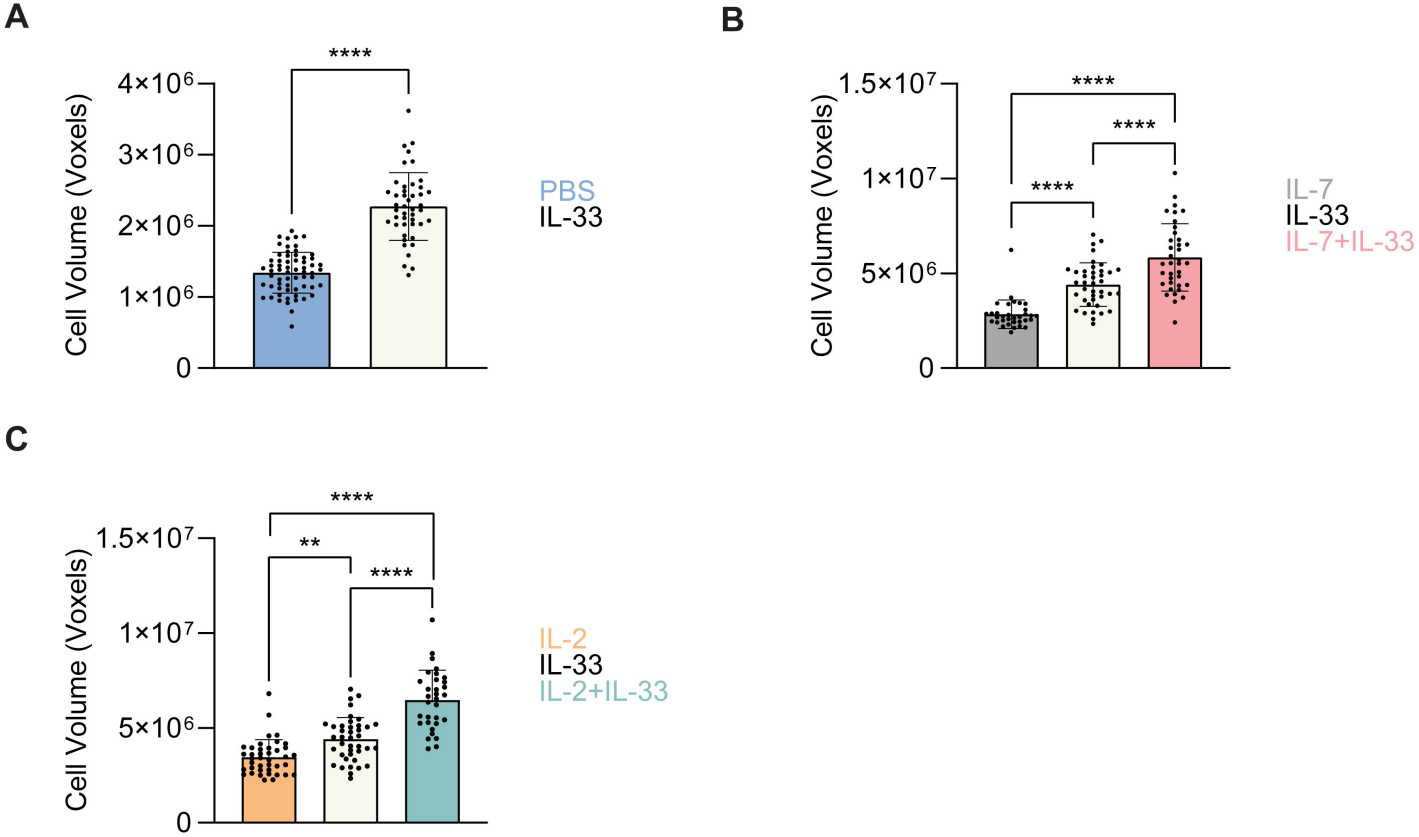
ILC2 increase in cell volume upon stimulation with activating cytokines. Quantification of the total cell volume using cytosolic BODIPY (493/503) fluorescence from confocal images. **(A)** group 2 innate lymphoid cells (ILC2) that underwent PBS intranasal challenge for three consecutive days are shown in blue (n = 30) and those treated with 250 ng/mL of IL-33 are shown in off-white (n = 33). **(B)** ILC2 were treated with either IL-7 (grey, n = 30), IL-33 (off-white, n = 30), or IL-7+IL-33 (pink, n = 30) for 24 hours prior to imaging. **(C)** ILC2 were treated with either IL-2 (orange, n = 30), IL-33 (off-white, n = 30), or IL-2+IL-33 (teal, n = 30) for 24 hours prior to imaging. All cytokines were administered at 10 ng/mL. Data are represented as average ± standard deviation (SD) where n = number of cells. The data representing the IL-33 stimulation is the same for **(B, C)**. **(A)** Statistical significance (*p*-values) was calculated using unpaired two-tailed Student’s t-test. **(B, C)** Statistical significance (*p*-values) was determined by applying ordinary one-way ANOVA and *post-hoc* Tukey’s multiple comparison tests (p < 0.01 = ** and p < 0.0001 = ****).

The cells from which the neutral LD analysis was performed in Sections 3.4 and 3.5 were also utilized for cell volume analysis. At steady state (PBS control treatment), *ex vivo* lung-derived ILC2 were shown to exhibit a very small cell volume which significantly increased when treated with IL-33 (**Figure 10A**). To further understand how cytokines known to drive ILC2 effector functions regulate cell volume, we analyzed *in vitro* bone marrow-derived ILC2 in the same conditions outlined in previous sections (Sections 3.1–3.5). Similarly to the *ex vivo* lung-derived ILC2, the cell volume presented by *in vitro* bone marrow-derived ILC2 treated with IL-7 alone were significantly smaller in comparison to all other cytokine treatments (**Figure 10B**). A dramatic increase in cell volume was observed in the presence of IL-33 alone, as well as with the combined treatment of IL-7+IL-33 (**Figure 10B**). This trend held true for ILC2 treated with IL-2 alone, which were significantly smaller in volume compared to all other cytokine treatments (**Figure 10C**). Concurrently, a significant increase in cell volume was observed in the presence of IL-33 alone, and with the combined treatment of IL-2+IL-33 (**Figure 10C**). In both cases the cell volume of either IL-7 alone or IL-2 alone doubled or nearly doubled when combined with IL-33. These data clearly demonstrate that cell volume is inextricably linked to ILC2 activation.

### 3.7 Neutral lipid droplet volume, number, and cell volume are correlated in group 2 innate lymphoid cells

There are many metrics that can be used to describe the diversity of LDs such as: volume, number, shape, and the size of the cell in which they reside. Large volumes of LDs have been correlated with large cell sizes in adipocytes (51) and the volume of LDs has been shown to correlate with the number of LDs in fibroblasts (52). We established in Section 3.5 that the distribution of neutral LD morphology is specific to different cytokine stimulations, but the intricacies of the relationships between these metrics have been unexplored in ILC2.

To investigate the potential relationships between cell volume, total lipid volume, and number of LDs, we performed correlation analysis between each of these metrics for each cytokine stimulation (**Table 4**). The data from Sections 3.4 – 3.6 were collated such that each cell volume corresponded with a total neutral lipid volume and neutral LD number. The strongest correlation among all the tested conditions was between the total volume of neutral lipids and the number of neutral LDs. Both IL-2 alone and IL-2+IL-33 exhibited a strong correlation between total volume and the number of neutral LDs, whereas IL-7 alone and IL-7+IL-33 exhibited a moderate correlation (**Table 4**). These data suggest that the synergy between IL-2 and IL-33 have the greatest impact on how neutral lipids are stored, as suggested in Section 3.5 (**Table 3**).

**Table 4:** Correlation analysis between morphology metrics.

Cytokine Treatment	LD Volume by LD Number	LD Volume by Cell Volume	LD Number by Cell Volume
*ex vivo* PBS	0.959*	0.479	0.461
*ex vivo* IL-33	0.081	0.348	0.063
IL-7	0.569	0.033	0.353
IL-2	0.773	0.606	0.532
IL-33	0.275	0.193	0.352
IL-7+IL-33	0.511	0.714	0.412
IL-2+IL-33	0.766	-0.11	-0.075

The values for total neutral lipid droplet (LD) volume per cell, total number of neutral lipid droplets per cell, and the volume of each cell were used to calculate correlating factors. Each cell is color-coded to distinguish significant correlation coefficients where grey shows no correlation (below 0.3), red indicates a low correlation (0.3-0.5), yellow is moderately correlated (0.5-0.7), and green is highly correlated (0.7-0.9). Values above 0.9 are very highly correlated and indicated with an asterisk.

The *ex vivo* lung-derived ILC2 at steady state (PBS) exhibited the strongest correlation between the total volume and the number of neutral LDs, where the low volume of neutral lipids and low number of droplets was very consistent (**Figure 7**). IL-7+IL-33 presented with the strongest correlation between total volume of neutral lipids and the cell volume, where there were consistently high volumes of neutral lipids and large cell volumes (**Figures 8**, **10**). A moderate correlation between total neutral lipid volume and cell volume was observed in IL-2 alone, whereas the *ex vivo* lung-derived ILC2 at both steady state (PBS) and with IL-33 activation exhibited a low correlation. Nearly every cytokine stimulation including the *ex vivo* naïve lung-derived ILC2 at steady state showed a low correlation between the number of neutral LDs and the cell volume (**Table 4**). These findings show that depending on how much neutral lipid content is present, ILC2 will selectively distribute their neutral lipid storage regardless of cytokine stimulation.

## 4 Discussion

ILC2 are vital to tissue barrier integrity, providing immunological protection by orchestrating innate and adaptive immune process as well as wound healing and tissue restoration. Recent data has implicated metabolic factors, such as lipid metabolism, that influence the role of ILC2 in airway inflammation and lung homeostasis (32, 33). The interplay of the immune system and lipid metabolism is highly complex, and the rarity of ILC2 *in situ* demands improved methodologies to study the intricacies of these relationships during type 2 immune responses and its associated pathologies. Here, we investigated the exogenous uptake of fatty acids (FAs), the transient storage of those FAs in neutral lipid droplets (LDs), and the utilization of FAs by mitochondrial fatty acid β-oxidation (FAO) for energy production to elucidate the phenotypes and effector functions associated with ILC2 lipid metabolism.

Our novel methods described here demonstrate that the utilization of lipid metabolism is markedly elevated upon ILC2 activation, revealing consistent findings comparing lung- and bone marrow-derived ILC2, as well as *ex vivo* and *in vitro* cultures. Our study reveals that ILC2 take up minimal levels of FAs, harbor low quantities of neutral LDs, and utilize FAO at a minimum rate during steady state (PBS controls, or stimulations with IL-7, or IL-2 only), but gradually increase lipid metabolism upon activation with IL-33 regardless of ILC2 origin. Concurrently, ILC2 from various tissues have been observed to take up FA at steady state (32) and were shown to considerably increase FA uptake upon activation (33, 34). Although the acquisition of *ex vivo* samples is necessary to capture the parameters of lipid metabolism during ILC2-mediated airway inflammation, the cost of acquiring these samples is high and detailed implications of single cytokine stimulations cannot be deciphered. We therefore made use of our efficient *in vitro* cell culture models of primary ILC2 (41), and our findings align well with previously reported observations demonstrating that FA uptake and lipid storage increases in ILC2 stimulated by IL-7+IL-33 (33). However, our study extends these findings, demonstrating increased FA uptake and storage upon stimulation with IL-2 alone as well as with the combination of IL-2+IL-33. Biologically, FAs are important for ILC2 proliferation and effector functions (33, 35, 53). Cell proliferation has a high energy demand; with increased proliferation following ILC2 activation we observe an increase in FA uptake, cell volume, and FAO.

A previous study demonstrated that cells prioritize phospholipid synthesis in the presence of glucose (54). In parallel, another study traced phospholipids in the membrane of activated ILC2 from exogenously acquired FAs (33). It is likely that in the early stages of ILC2 activation when glucose availability is high, FAs are utilized first for phospholipid synthesis resulting in a larger cell volume. As glucose levels decline, ILC2 may begin to breakdown stored or exogenous FA via β-oxidation as an energy source. In fact, during helminth infection, it has been reported that ILC2 metabolize exogenous FAs via FAO to fuel oxidative phosphorylation, which is necessary for ILC2 proliferation and cytokine production (32). Our study corroborates this data, supporting the notion that cell proliferation, cytokine production, FA uptake and storage, and FAO activity are all inextricably linked with ILC2 activation. The combination of IL-7 or IL-2 with IL-33 displayed the strongest increase in FA uptake and FAO. It is likely that this increase in FAO among strongly activated ILC2 provides a source of acetyl-CoA to help fuel the tricarboxylic acid (TCA) cycle and a source of NADH and FADH_2_ to in turn fuel the respiratory chain, subsequently promoting pro-inflammatory ILC2 activity (53). Although there are other methods to measure FAO, such as the tracing of radiolabeled FAs, the Agilent Seahorse XF Palmitate Oxidation Stress Test, targeted gene knockouts, or the pharmacological inhibition of carnitine palmitoyl transferases (CPTs), our implementation of FAO-Blue provides a more feasible and rapidly accessible flow cytometry-based method for measuring FAO levels in ILC2. This innovative assay allows analysis of FAO levels in small and heterogenous *ex vivo*-isolated cell populations or limiting *in vitro* cell culture systems and opens the door for future studies where the effect of FAO inhibitors or nutrient availability could be directly measured using FAO-Blue.

Neutral lipids are packaged in LDs, which is driven by lipid neogenesis, exogenous acquisition of FAs, or internal recycling of FAs and are transiently stored in the form of triglycerides (TGs) (45). It has been recently shown that LDs help drive pathogenic airway inflammation mediated by ILC2 (33). Although the effect of IL-33 or allergen intranasal challenge on lipid storage in ILC2 has been thoroughly investigated using flow cytometry, the representation of intracellular lipid storage has been shown almost exclusively using qualitative assessments (33, 34). Hence, we employed here microscopy to quantify how neutral lipids are stored in droplets, demonstrating that each cytokine treatment induced a unique LD distribution phenotype that could only be discovered through visual intracellular investigation. While in flow cytometry, ILC2 treated with IL-7 or IL-2 alone both harbor low levels of stored lipids, IL-7 uniquely has the fewest number of LDs overall with the largest proportion of small-sized LDs (57%). ILC2 stimulated with IL-7 or IL-2 in combination with IL-33 also share similarity in their high levels of stored lipids measured through flow cytometry, however, the latter has the smallest proportion of small-sized LDs at only 27%. This is a significant shift from its steady state condition in IL-2 alone where 51% of the LD population were small-sized. The most common and strongest correlation found was between the total volume of stored lipids and the number of LDs. This suggests that ILC2 distribute their lipid storage between many droplets as opposed to several extremely large droplets either due to cytoplasmic restrictions or to accommodate distinct metabolic requirements.

Indeed, a previous study demonstrated that during nutrient deprivation, LDs undergo dispersion along microtubules to efficiently supply FAs for β-oxidation in mitochondria (54). As LDs are formed as an energy reserve, it is possible that variations in nutrient accessibility during the period of ILC2 stimulation directly impacts the distribution of LD size, which remains to be explored in future studies.

Taken together this data suggests that as ILC2 are being strongly activated they acquire large levels of exogenous FAs that are used for FAO, and any excess FAs are stored in LDs to avoid lipotoxicity resulting in large-sized LDs. Furthermore, small-sized LDs are likely degraded before large-sized LDs through lipolysis or lipophagy to create an easily accessible pool of free FA to fuel FAO when energy demands are high. Interestingly, we observed that the sphericity of LDs in ILC2 shifts from being spherical when stimulated IL-2 alone to being more irregularly-shaped following activation with IL-33. This phenomenon corresponds with observation of a study demonstrating that marked changes in LD morphology are due to an increased number of contact sites with mitochondria (54). The consistently large proportion of intermediate-sized LDs among highly activated ILC2 could have several explanations as the transition states of small to large-sized LDs is likely a highly dynamic process. Potentially, small LDs may be catabolized by lipolysis into FAs that are immediately stored again into pre-existing small LDs alongside exogenously acquired FAs to form intermediate-sized LDs. Conversely, these intermediate LDs could be formed through the degradation of large-sized LDs into smaller volumes. The dynamic nature of this process could be further investigated through the implementation of live imaging, where LD size transition could be recorded in real time to explain these morphological and distribution differences. Moreover, kinetic experiments involving earlier time points or live imaging of LDs in tandem with other organelles, such as lysosomes or mitochondria, could explain these morphological and distribution differences, and could provide further in-depth insights of the lipid metabolism in ILC2.

## Data Availability

The raw data supporting the conclusions of this article will be made available by the authors, without undue reservation.

## Supplementary material

Supplementary Figure 1 Gating strategies for the sorting of murine bone marrow-derived ILC2 progenitors and lung ILC2. **(A)** After the exclusion of debris and doublets, murine bone marrow-derived group 2 innate lymphoid cells (ILC2) precursors were defined and isolated by flow cytometric sorting as lineage-negative, c-kit^-^Sca-1^+^CD25^+^ cells. **(B)** After excluding dead cells, debris and doublets, murine lung ILC2s were isolated by flow cytometric sorting as lineage-negative, CD45^+^Thy-1.2^+^ST2^+^CD25^+^ cells.
