# Supplementary figures and images for: Analysis of lipid uptake, storage, and fatty acid oxidation by group 2 innate lymphoid cells
Source: Front Immunol. 2024 Oct 21;15:1493848. doi: 10.3389/fimmu.2024.1493848 (PMC11532145; doi:10.3389/fimmu.2024.1493848)

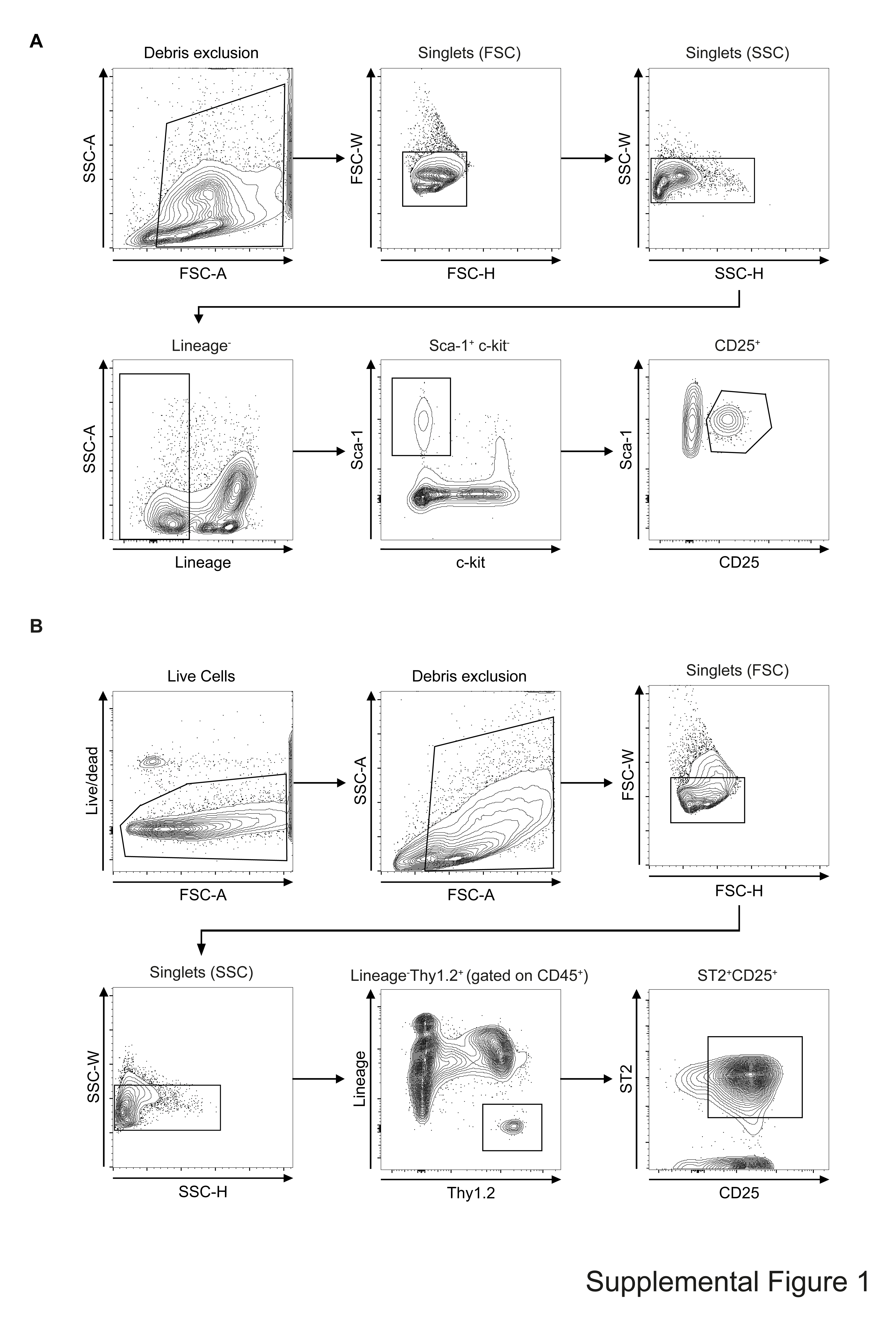

Supplement: Supplementary Figure 1 — Gating strategies for the sorting of murine bone marrow-derived ILC2 progenitors and lung ILC2. (A) After the exclusion of debris and doublets, murine bone marrow-derived group 2 innate lymphoid cells (ILC2) precursors were defined and isolated by flow cytometric sorting as lineage-negative, c-kit-Sca-1+CD25+ cells. (B) After excluding dead cells, debris and doublets, murine lung ILC2s were isolated by flow cytometric sorting as lineage-negative, CD45+Thy-1.2+ST2+CD25+ cells. [file Image1.jpeg]
